# Human umbilical cord-derived mesenchymal stem cells ameliorate non-alcoholic fatty liver disease via activating TFEB-mediated autophagy in male mice

**DOI:** 10.1186/s13287-025-04855-9

**Published:** 2025-12-13

**Authors:** Huina Zhang, Peng Liu, Yaxuan Deng, Li Wu, Orion Fan, Yanling Cui, Chunxue Zhang, Wenmin Zhu, Yi Eve Sun, Chuwen Lin, Congrong Wang

**Affiliations:** 1 Department of Endocrinology & Metabolism, Shanghai Fourth People’s Hospital, School of Medicine, Tongji University, Shanghai, 200434 China; 2 Stem Cell Translational Research Center, Tongji Hospital, School of Medicine, Tongji University, Shanghai, 200065 China; 3 Department of Cardiology, Shanghai Ninth People’s Hospital, Shanghai Jiao Tong University School of Medicine, Shanghai, 200011 China; 4 Shanghai Institute of Stem Cell Research and Clinical Translation, Shanghai East Hospital, School of Medicine, Tongji University, Shanghai, 200120 China

**Keywords:** NAFLD, Autophagy, hUC-MSCs, Transcription factor EB

## Abstract

**Background:**

Non-alcoholic fatty liver disease (NAFLD) is characterized by abnormal lipid accumulation in hepatocytes and defective autophagy has been implicated in its pathogenesis. Human umbilical cord-derived MSCs (hUC-MSCs) have shown therapeutic potential in treating NAFLD, while underlying molecular mechanisms remained largely unknown.

**Methods:**

Male C57BL/6J mice fed a choline-deficient high fat diet (CD-HFD) and HepG2 cells exposed to palmitic acid/oleic acid were established as in vivo and in vitro models of NAFLD, respectively. Both models were subjected to treatment with human umbilical cord-derived MSCs (hUC-MSCs). Lipid content, proinflammatory cytokines, fibrosis markers and the hepatic transcriptome were assessed to determine the effect of hUC-MSCs.

**Results:**

Here, hUC-MSCs decreased hepatic lipid content and alanine aminotransferase/aspartate aminotransferase levels, as well as attenuated inflammation and fibrosis in choline-deficient high-fat diet (CD-HFD)-induced NAFLD mice. Mechanistically, hUC-MSCs restored impaired autophagic flux and mitigated liver steatosis through the AMPK-mTOR-TFEB pathway in both NAFLD mice and oleic acid/palmitic acid-induced “fatty” HepG2 cells. Of note, hUC-MSCs have been found to promote nuclear translocation of TFEB in PA/OA-induced HepG2 cells. Additionally, TFEB knockdown partially attenuated the effect of hUC-MSCs on enhancing autophagy and lipid metabolism in vitro.

**Conclusions:**

This study suggests that hUC-MSCs represent a potential therapeutic approach to treating NAFLD through activating TFEB-mediated autophagy.

**Supplementary Information:**

The online version contains supplementary material available at 10.1186/s13287-025-04855-9.

## Introduction

Non-alcoholic fatty liver disease (NAFLD) is the most common chronic liver disease, with a global prevalence reaching 32.4% [1]. The disease spectrum of NAFLD encompasses non-alcoholic fatty liver, non-alcoholic steatohepatitis (NASH), liver cirrhosis, and even hepatocellular carcinoma. Furthermore, NAFLD is closely associated with diabetes and cardiovascular diseases, posing a significant threat to human health. Developing effective therapies for NAFLD has proven to be a challenge due to the complexity of the disease. An ideal treatment for NAFLD should effectively target fat accumulation, inflammation, and fibrosis while maintaining a robust safety profile for long-term use. Despite substantial research efforts, no existing pharmacological intervention has fully satisfied these criteria. For many years, the primary management strategy for NAFLD has focused on lifestyle modifications and weight loss. Although bariatric surgery has shown therapeutic effects for NAFLD [2, 3], its effectiveness is frequently compromised by challenges related to patient compliance and the limited availability of comprehensive clinical evidence supporting its long-term benefits. Recently, the Food and Drug Administration (FDA) approved resmetirom, an oral thyroid hormone receptor-beta (THRβ) agonist, as a treatment for adult patients with NASH and associated fibrosis [4]. In clinical trials, resmetirom demonstrated a significant benefit in halting fibrosis progression. However, this effect was observed in only a subset of patients (approximately 25–30%) [5], emphasizing the urgent need to explore and develop innovative therapeutic approaches for NAFLD.

Emerging evidence has shown that mesenchymal stem cells (MSCs) could be a promising therapeutic strategy for NAFLD treatment [6, 7]. MSCs comprise a subset of stromal stem cells with the capacity for self-renewal and multidirectional differentiation. MSCs can be isolated from a variety of tissues and organs, including adipose tissue, bone marrow, umbilical cord, placenta, and cartilage. Compared with MSCs from other sources, human umbilical cord-derived mesenchymal stem cells (hUC-MSCs) have demonstrated advantages due to their non-invasive accessibility, higher proliferative capacity, and lower expression of human leukocyte antigen. It has been suggested that multiple infusions of hUC-MSCs via tail vein could alleviate steatosis, inflammation, and fibrosis of NASH in mouse models [8–10]. Additionally, hUC-MSCs in combination with liraglutide significantly inhibited the expression of cytokines and ameliorated liver injury [11]. However, the underlying molecular mechanisms have remained largely unknown.

Autophagy is a process that delivers damaged organelles and misfolded proteins to lysosomes for degradation, which helps to maintain cellular homeostasis. Autophagy has been shown to play an important role in the pathogenesis of NAFLD [12]. Previous research has found that autophagic flux is impaired in the livers of NAFLD patients [13, 14]. In vitro studies have demonstrated that blockage of hepatic autophagy, reduced lysosomal degradation for lipogenic enzymes, and increased triglyceride synthesis, leading to hepatic steatosis. Therefore, targeting autophagy could be a therapeutic strategy for the treatment of NAFLD. Existing literature has suggested that MSCs protect the liver against ischemia/reperfusion injury via enhancing autophagy [15]. Nevertheless, how hUC-MSCs regulate autophagy in NAFLD has not been elucidated.

The transcription factor EB (TFEB) is a key regulator of autophagy and lysosomal biogenesis. The activity of TFEB is related to its phosphorylation status and cellular distribution. Dephosphorylation of TFEB promotes its nuclear translocation, thereby activating the transcription of autophagy-related genes [16]. It has been reported that nuclear TFEB expression is inversely correlated with the steatosis severity of liver biopsy samples from NAFLD patients [17]. Moreover, liver specific TFEB knockout mice presented with impairment of lipid degradation as well as fatty acid oxidation [18, 19]. Conversely, TFEB overexpression decreased lipid droplet levels and weight gain induced by high fat diet [20]. These studies highlight the importance of TFEB in regulating NAFLD. However, whether TFEB mediates the therapeutic effects of hUC-MSCs on NAFLD remains to be determined.

In this study, the mechanistic role of autophagy in the hUC-MSC-mediated treatment of NAFLD was investigated. In vivo, hUC-MSCs attenuated hepatic steatosis, inflammation, and fibrosis in NAFLD mouse models through the activation of autophagy. Moreover, knockdown of TFEB reduced the therapeutic effects of hUC-MSCs on NAFLD. These results further reinforce the pivotal role of autophagy in the treatment of NAFLD and highlight the therapeutic potential of hUC-MSCs as an effective strategy against this disease.

## Materials and methods

### Experimental animals

All relevant procedures involving animal experiments were in compliance with the guidelines of the Animal Welfare Ethics Committee of Shanghai Sixth People’s Hospital. Eight-week-old male C57BL/6J mice were purchased from Shanghai SLAC Laboratory Animal Co. All mice were kept in a 12 h light/dark cycle with free access to food and water. The experimental protocols complied with the ARRIVE guidelines 2.0 (Animal Research: Reporting of In Vivo Experiments).

### hUC-MSCs culture

The hUC-MSCs were obtained from the Translational Medical Center for Stem Cell Therapy at Shanghai East Hospital. The experimental protocol was approved by the Ethics Committee of Shanghai East Hospital. Clinical-grade hUC-MSCs were produced in accordance with good manufacturing practices (GMP) standards at Shanghai East Hospital. The protocol for hUC-MSC isolation and culture was performed as previously described in our study [21]. hUC-MSCs at passage 4 were collected for intravenous injection. All cell preparations tested negative for mycoplasma contamination.

### Animal treatment

After one month of adaptive feeding, the mice were randomly assigned to two groups and fed either a standard diet (Control) or a choline-deficient high-fat diet (CD-HFD) (44.9 kcal% fat, 35.1 kcal% carbohydrates and 20.0 kcal% protein, without added choline) (D05010402, Research Diets). Mice fed with the CD-HFD showed significant weight gain after 16 weeks of experimental feeding. Subsequently, the CD-HFD mice were randomly divided into two groups: one group received 1 × 10^6^ hUC-MSCs suspended in 0.2 ml phosphate-buffered saline (PBS, Cytiva, SH30256.01) via tail vein injection (referred to as the CD-HFD hUC-MSCs group), while the other group received an equal volume of PBS alone (referred to as the CD-HFD group). The hUC-MSCs were administered every two weeks for a total of five injections. The weight of each mouse was measured at the onset of the diet and on the day of euthanasia. All mice were humanely euthanized at week 26 of the CD-HFD feeding regimen, one week after the fifth hUC-MSC injection. During the hUC-MSC treatment period, the mice continued receiving the CD-HFD. After overnight fasting, mice were anesthetized with isoflurane. Subsequently, blood was collected via cardiac puncture. After blood collection, the mice were euthanized by cervical dislocation, and liver and other tissue samples were collected and stored at − 80℃.

### In vivo imaging of fluorescently labeled hUC-MSCs

To investigate the biodistribution of hUC-MSCs, living mice with non-alcoholic fatty liver disease were injected with DiR-labeled hUC-MSCs. Briefly, the DiR dye (Invitrogen, D12731) was thoroughly mixed with hUC-MSC suspension and incubated at 37 ℃ for 30 min. Subsequently, the cells were washed twice with PBS to eliminate excess dye. The DiR-labelled hUC-MSCs (1 × 10^6^ cells) were then suspended in 0.2 ml PBS and injected into CD-HFD mice via the tail vein. The standard diet control group received an equivalent volume of PBS. Imaging was performed at 1, 7, and 14 days after cell infusion using the IVIS^®^Spectrum Imaging System (PerkinElmer).

### Histological analysis

For histological analysis, fresh liver specimens were fixed in 4% paraformaldehyde (PFA, Sangon Biotech) at 4 °C overnight and embedded in paraffin. Paraffin section (5 μm) were stained with hematoxylin and eosin (H&E, Sigma) according to the standard protocol. The morphological structure of each tissue was examined under a light microscope.

### Oil red O staining

To observe hepatic steatosis, liver tissues were embedded in Tissue-Tek O.C.T. compound (Sakura) and stored at − 80℃ until sectioning. Tissue blocks were cut into 10 μm sections and then stained with Oil Red O (Sigma-Aldrich, O0625), followed by counterstaining with hematoxylin according to the manufacturer’s instructions.

For in vitro studies, HepG2 cells were washed three times with PBS and fixed in 4% PFA for 20 min. The fixed cells were stained with an Oil Red O Staining Kit (Beyotime, C0157) according to the manufacturer’s instructions. Images were acquired under a light microscope.

### Biochemical and lipid accumulation measurements

An automatic biochemistry analyzer (Rayto Technologies) was used to assess the levels of serum alanine aminotransferase (ALT) and aspartic acid transaminase (AST). The triglyceride (TG) levels of liver tissues and HepG2 cells were measured using an Enzymatic Assay Kit (Nanjing Jiancheng) according to the manufacturer’s protocol.

### Quantitative real-time reverse transcription PCR (qRT-PCR) analysis

Total RNA was isolated from liver tissues using TRIzol reagent (Invitrogen, 15596026) and reverse transcribed into cDNA with a reverse transcription kit (Vazyme, R323) according to the manufacturer’s instructions. Real-time PCR was performed on cDNA samples using 2×Taq Pro Universal SYBR qPCR Master Mix (Vazyme, Q712) with an ABI Prism 7300 Thermal Cycler (Applied Biosystems). The relative changes in gene expression were determined using the formula 2-ΔΔCt. Primers used for gene expression are listed in Supplementary material 3.

### Western blot analysis

Cells and liver tissues were lysed in ice-cold RIPA buffer (Beyotime, P0013). The protein concentrations were determined using a BCA Protein Assay Kit (Beyotime, P0009). Equal amounts of total protein were electrophoresed with 10% SDS-polyacrylamide gel (SDS-PAGE) (Epizyme Biotech) and transferred to a polyvinylidene fluoride (PVDF) membrane (Merck Millipore). The membranes were blocked with 5% non-fat milk (BD Difco) for 1 h at room temperature and immunoblotted using polyclonal primary antibodies against SREBP-1 (1:1000, Invitrogen), a-SMA (1:1000, Proteintech), FASN (1:1000, Abcam), COL1A1 (1:1000, Sigma), p-mTOR (1:1000, Abcam), mTOR (1:1000, Abcam), AMPK (1:1000, Cell Signaling Technology), p-AMPK (1:1000, Cell Signaling Technology), TFEB (1:1000, Cell Signaling Technology), LC3B (1:1000, Abcam), GAPDH (1:10 000, Proteintech) and Histone-H3 (1:2000, Abcam). The immunoblots were visualized with a 1:50,000 dilution of horseradish peroxidase (HRP)-conjugated polyclonal secondary antibody (Cell Signaling Technology) and an enhanced chemiluminescence (ECL) detection kit (Abclonal). ImageJ software (NIH) was used for densitometric analysis of the bands.

### Enzyme-linked immunosorbent assay (ELISA)

The levels of IL-6 were measured using Mouse IL-6 High Sensitivity ELISA Kit according to the manufacturer’s instructions (Multi Sciences, EK206HS-96). Mouse IL-1β ELISA Kit (Multi Sciences, EK201B/3–96), Human IL-1β ELISA Kit (Multi Sciences, EK101B), and Human/Mouse/Rat TGF-β1 ELISA Kit (Multi Sciences, EK981-96) were used to detect the levels of IL-1β and TGF-β1 respectively.

### RNA-seq analysis

Total RNA was extracted from liver tissue using TRIzol^®^ Reagent (Invitrogen) according to the manufacturer’s instructions. Then RNA quality was determined by 5300 Bioanalyzer (Agilent) and quantified using the ND-2000 (NanoDrop Technologies). The liver RNA-seq transcriptome library was prepared following Illumina^®^ Stranded mRNA Prep, Ligation (San Diego, CA) using 1 µg of total RNA. The sequencing library, after quantification with a Qubit 4.0 fluorometer, was sequenced on the NovaSeq X Plus platform (PE150) using a NovaSeq Reagent Kit at Shanghai Majorbio Bio-pharm Biotechnology Co., Ltd. (Shanghai, China). The raw paired-end reads were trimmed and quality-checked using fastp [22] with default parameters. The clean reads were then aligned to the reference genome using HISAT2 [23]. Subsequently, the aligned reads were assembled in a reference-guided manner by StringTie [24].

### Identification of differentially expressed genes (DEGs)

Differential gene expression analysis was performed using the DESeq2 package in R. DEGs between the CD-HFD hUC-MSC-treated and CD-HFD vehicle groups were identified using the results function with the false discovery rate (FDR)-adjusted *p*-values. Genes with an FDR-adjusted *p*-value < 0.05 and absolute log2 fold change ≥ 0.5 were considered significantly differentially expressed. Genes with log2 fold change > 0.5 were classified as upregulated, and those with log2 fold change < − 0.5 were classified as downregulated. Genes not meeting these criteria were considered stable.

### Functional enrichment analysis

Functional enrichment analysis of the identified DEGs was performed using the clusterProfiler R package. GO enrichment analysis was conducted to investigate the biological processes (BP), cellular components (CC), and molecular functions (MF) associated with DEGs, although only the BP were presented in this study. KEGG pathway enrichment analysis was performed to identify significantly enriched signaling pathways. Terms and pathways with an FDR-adjusted *p*-value < 0.05 were considered statistically significant.

### Isolation of cytosolic and nuclear proteins

The cytosolic and nuclear proteins were isolated using a commercial kit (Thermo Scientific, 78833) according to the manufacturer’s instructions. The extracts were stored at − 80 °C until use.

### Culture and treatment of HepG2 cells

HepG2 cells were cultured in DMEM High Glucose (Cytiva, SH30243.01) supplemented with 10% fetal bovine serum (Gibco), 100 U/ml penicillin, and 100 µg/ml streptomycin (Gibco) at 37 °C in a humidified incubator containing 5% CO_2_. The medium was changed every 2 days. Cells were passaged at a 1:4 ratio when they reached 80% confluency. HepG2 cells were stimulated with oleic acid (OA) (Sigma, O-7501) and palmitic acid (PA) (Sigma, P-9767) to establish an in vitro model of NAFLD. The cells were stimulated with OA (200 µM) and PA (100 µM) or BSA (Sigma, B-2064) control for 24–48 h.

After PA/OA treatment, the cells were then cultured in fresh standard medium (DMEM without PA/OA) and treated with or without hUC-MSCs or 3-MA (Selleck, S2767, 5 mM) for 12 h.

For plasmid transfection experiments, HepG2 cells were transfected with plasmids for 24 h using the LipoFiterTM Liposomal Transfection Reagent (Hanbio) according to the manufacturer’s instructions, before OA and PA treatment. The pCMV-mCherry-GFP-LC3B plasmid was purchased from Beyotime, the pCDH-mRFP-EGFP-LC3B plasmid from Miaoling Biology, and the TFEB-GFP plasmid from Asia-Vector Biotechnology (Shanghai) Co., Ltd.

The cells were fixed in 4% PFA for 15 min at room temperature. After being washed three times with PBS, cell nuclei were stained with DAPI (Roche) for 10 min and then washed three more times with PBS. The cells were visualized using a super-resolution confocal laser scanning microscope (Carl Zeiss).

### Lentiviral transfection

To further validate the TFEB knockdown phenotype in HepG2 cells, a synthetic human TFEB shRNA plasmid was purchased from Shanghai Genechem. The TFEB shRNA target sequence is 5′-CCCCTCATTACCAGTGAAGGA-3′. GV248-puro shRNA vectors targeting TFEB or a scrambled control (shRNA-NC) were co-transfected into HEK293T cells together with the lentiviral packaging plasmid pCMVΔ8.91 and envelope plasmid pCMV-*VSV*-*G* using LipoFiterTM Liposomal Transfection Reagent (Hanbio) according to the manufacturer’s instructions. After 48 h of incubation, the supernatant containing virus particles was collected and filtered through a 0.45 μm membrane filter. Subsequently, 500 µl to 1 ml of viral supernatant was added to a 60 mm dish to infect HepG2 cells. After 24 h of incubation, the culture medium was replaced with fresh medium, and the cells were further cultivated for 48 h. The medium was then replaced with 0.5 µg/ml of puromycin to select cells successfully infected with the virus.

### Statistical analysis

Statistical analysis was performed using Graphpad Prism software. The data were expressed as the mean ± SD. Data were analyzed using Student t-test and one-way analysis of variance with Tukey’s multiple comparisons test. *p-*value < 0.05 was considered statistically significant.

## Results

### Bio-distribution of hUC-MSCs in NAFLD mice

**Fig. 1:**
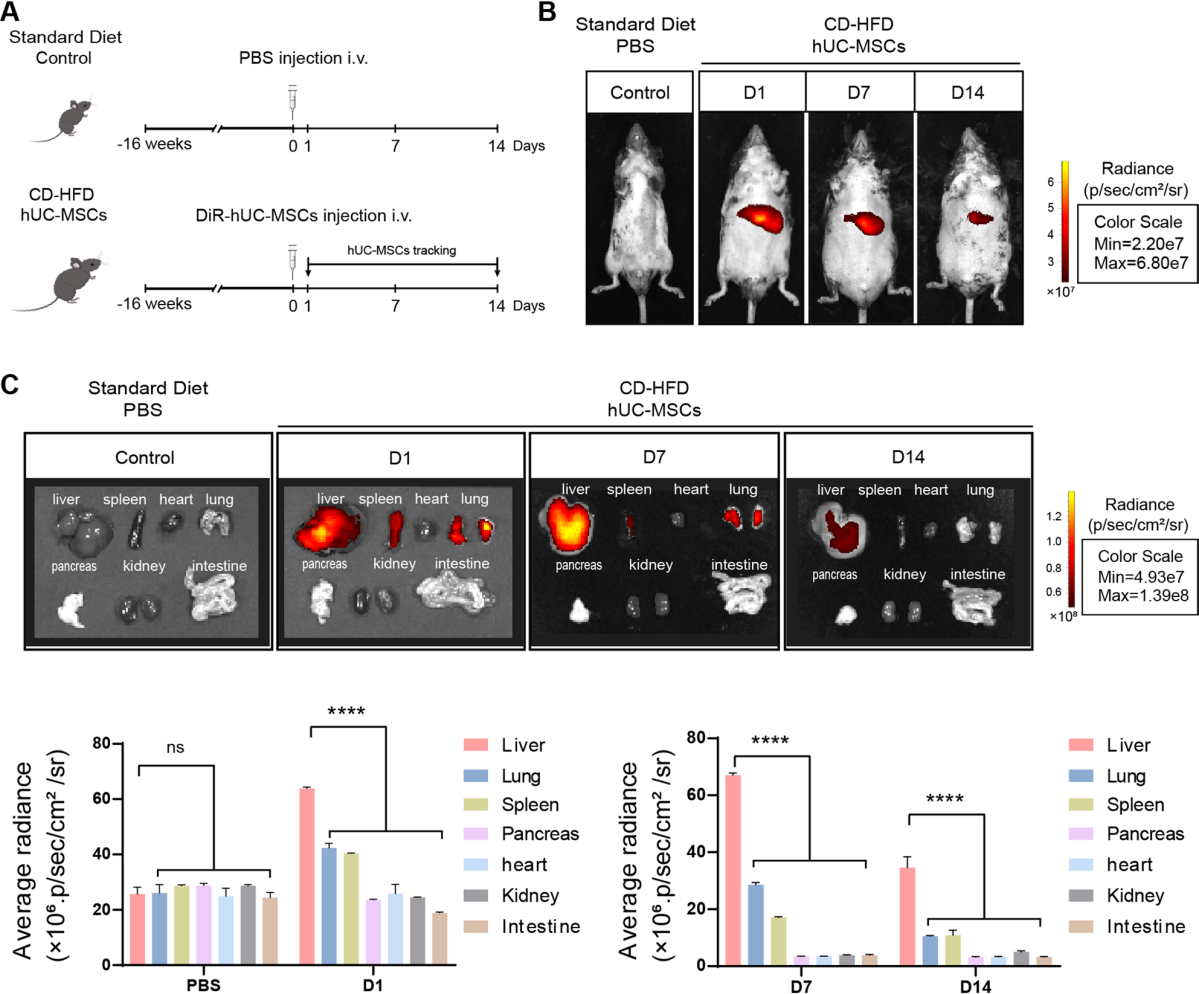
Biodistribution of hUC-MSCs after intravenous infusion into choline-deficient high-fat diet (CD-HFD)-induced NAFLD mice. **A** Study design for hUC-MSC bio-distribution. **B** Representative fluorescence images of in vivo tracking of DiR-labeled hUC-MSCs at day 1, 7 and 14. **C** Representative ex vivo fluorescence images of DiR-labeled hUC-MSCs from mouse organs, including the liver, spleen, heart, lung, pancreas, kidney, and intestine. Imaging was performed using IVIS spectrum in vivo imaging system at the indicated time points. Control mice were fed a standard diet and treated with PBS. Data were expressed as mean ± SD, ****P < 0.0001

To establish NAFLD models, C57BL/6J mice were fed with CD-HFD for 16 weeks to induce hepatic steatosis (Fig. 1A). Next, we assessed whether hUC-MSCs preferentially accumulated in injured livers. The live NAFLD mice were intravenously injected with DiR-labeled hUC-MSCs and imaged at 1, 7, and 14 days using an in vivo imaging system (IVIS). From in vivo imaging, the fluorescence of DiR-labeled hUC-MSCs was detected at 24 h after administration and tapered gradually throughout 14 days in the CD-HFD hUC-MSCs group (Fig. 1B). Then, we evaluated the fluorescence intensity in all harvested organs of NAFLD mice treated with DiR-labeled hUC-MSCs. Significantly stronger fluorescent signals were observed on day 1 in the liver after DiR-labeled hUC-MSC injection compared with other tissues. This suggests that hUC-MSCs in circulation localized more in the livers in NAFLD mice. By day 14, fluorescence signals were predominantly localized in the liver, with minimal to no detectable signals in other organs (Fig. 1C). These results demonstrated that hUC-MSCs could home to the liver of NAFLD mice.

### hUC-MSC infusion reduced liver steatosis, inflammation, hepatotoxicity, and fibrosis in NAFLD mice

**Fig. 2:**
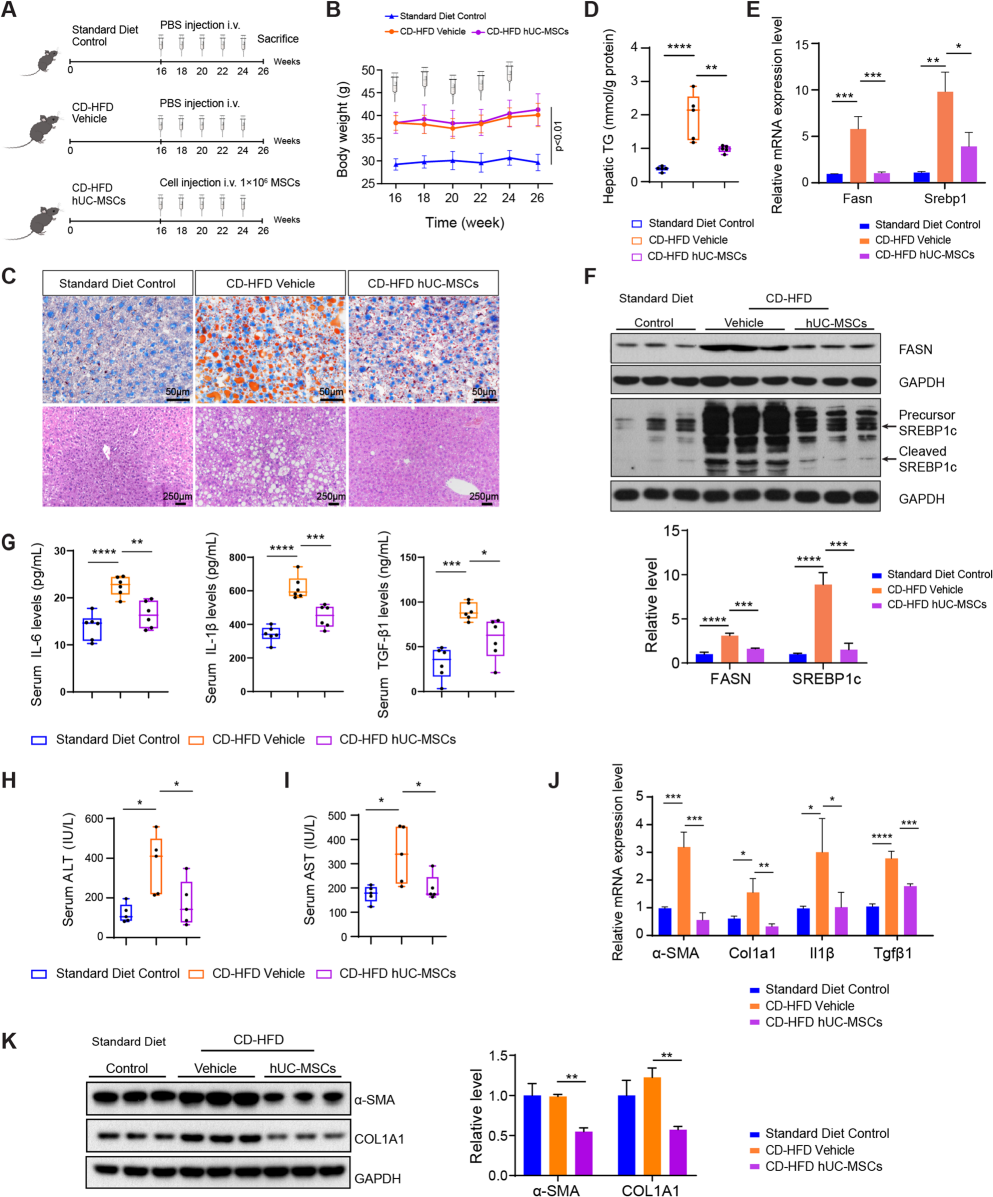
hUC-MSC infusion alleviated liver steatosis, inflammation, hepatotoxicity and fibrosis in choline-deficient high-fat diet (CD-HFD)-induced NAFLD mice. ** A** Schematic outline of research design for the hUC-MSC therapies in NAFLD mice. The 12-week-old C57BL/6J mice were fed with CD-HFD for 16 weeks and then treated with PBS or hUC-MSCs intravenously every 2 weeks for a total of 5 injections. Control mice were fed with a standard diet. **B** Weight changes of mice fed with a standard diet or CD-HFD over a 10-week period. Compared with mice fed with standard diet, mice fed with CD-HFD showed significantly elevated body weight. **C** Representative images of Oil Red O (400×) and hematoxylin-eosin (H&E) (200×) staining are shown for liver sections from mice in each group. Cell nuclei were stained with haematoxylin. **D** Hepatic triglycerides (TG) were measured after overnight fasting. **E**, **F** The mRNA and protein levels of lipogenic genes (FASN and SREBP1c) in the livers of mice were detected by quantitative real-time reverse transcription PCR (qRT-PCR) and western blot, respectively. **G** The concentrations of serum inflammatory cytokines (IL-6, IL-1β and TGF-β1) were detected by ELISA. **H**, **I** The levels of serum alanine aminotransferase (ALT) and aspartate aminotransferase (AST) were assessed after overnight fasting to evaluate liver function. **J** The mRNA levels of fibrosis-associated genes (α-SMA and Col1a1) and inflammation-related genes (Il1β and Tgfβ1) in the livers of mice were detected by qRT-PCR. **K** The level of fibrosis-associated proteins (α-SMA and COL1A1) in the livers of mice were detected by western blot. GAPDH serves as internal control. All full-length western blots are presented in Supplementary material 1: Fig. 2. *n* ≥ 3 per group. Data were expressed as mean ± SD, *P < 0.05, **P < 0.01, ***P < 0.001, ****P < 0.0001

The established NAFLD mice were randomized to either PBS (CD-HFD group) or hUC-MSC treatment (CD-HFD hUC-MSCs group) every 2 weeks for a total of 5 injections. Normal mice fed with a standard diet and treated with PBS served as controls (Fig. 2A).

NAFLD mice gained significantly more weight than standard diet controls, while hUC-MSC transplantation did not affect body weight (Fig. 2B). To assess whether hUC-MSC-based therapies could prevent liver steatosis in NAFLD mice, hematoxylin and eosin (H&E) as well as Oil Red O stains were employed to observe hepatic morphological changes and lipid deposition, respectively. As shown in H&E staining, NAFLD mice displayed enlarged hepatocytes, abundant lipid vacuoles and hepatocellular ballooning, while hUC-MSC treated mice presented smaller-sized hepatocytes with fewer intracellular lipid droplets (Fig. 2C). Additionally, Oil Red O staining revealed that hUC-MSC infusion reduced lipid droplet formation in the liver tissues of NAFLD mice, indicative of steatosis alleviation (Fig. 2C). Additional quantitative analysis confirmed that hUC-MSCs decreased hepatic TG accumulation in NAFLD mice (Fig. 2D). To further assess the effects of hUC-MSCs on lipogenesis, the expression profiles of genes related to hepatic fatty acid synthesis, including fatty acid synthase (FASN) and sterol regulatory element-binding protein-1c (SREBP1c), were analyzed. FASN is a key enzyme involved in de novo lipogenesis, directly contributing to the synthesis of fatty acids in the liver, which is particularly relevant in the context of NAFLD. SREBP1c acts as a master regulator of lipid homeostasis, controlling the expression of various genes associated with fatty acid and triglyceride metabolism [25, 26]. The expression profiles of SREBP1c and FASN at both the RNA and protein level were markedly upregulated in liver tissues from NAFLD mice compared to standard diet controls, whereas hUC-MSC intervention reversed the increase of SREBP1c and FASN (Fig. 2E and F). Thus, our results suggested that hUC-MSCs attenuated hepatic steatosis and reduced lipogenesis.

Hepatic inflammation, which causes liver damage and induces transition from steatosis to fibrosis, plays an essential role in promoting NAFLD progression [27]. Therefore, the effects of hUC-MSCs on inflammation, hepatotoxicity and fibrosis were examined. Compared with standard diet controls, NAFLD mice presented elevated expression of proinflammatory cytokines (IL-6, IL-1β and TGF-β1) as well as increased serum levels of ALT and AST, which indicated hepatic inflammation and impaired liver function. However, after hUC-MSC treatment, proinflammatory cytokines and liver functional markers were remarkably reduced, illustrating the protective effects of hUC-MSCs on inflammation and hepatotoxicity (Fig. 2G-J). Furthermore, the mRNA and protein levels of alpha-smooth muscle actin (α-SMA) and collagen type I alpha 1 (COL1A1) were determined to assess liver fibrosis. Both α-SMA and COL1A1 were markedly downregulated by hUC-MSC intervention compared with NAFLD mice (Fig. 2K). Taken together, these data demonstrated that hUC-MSCs protected NAFLD mice against liver inflammation, hepatotoxicity and fibrosis.

### hUC-MSC alleviated steatosis, inflammation, and fibrosis in PA/OA induced HepG2 cells

**Fig. 3:**
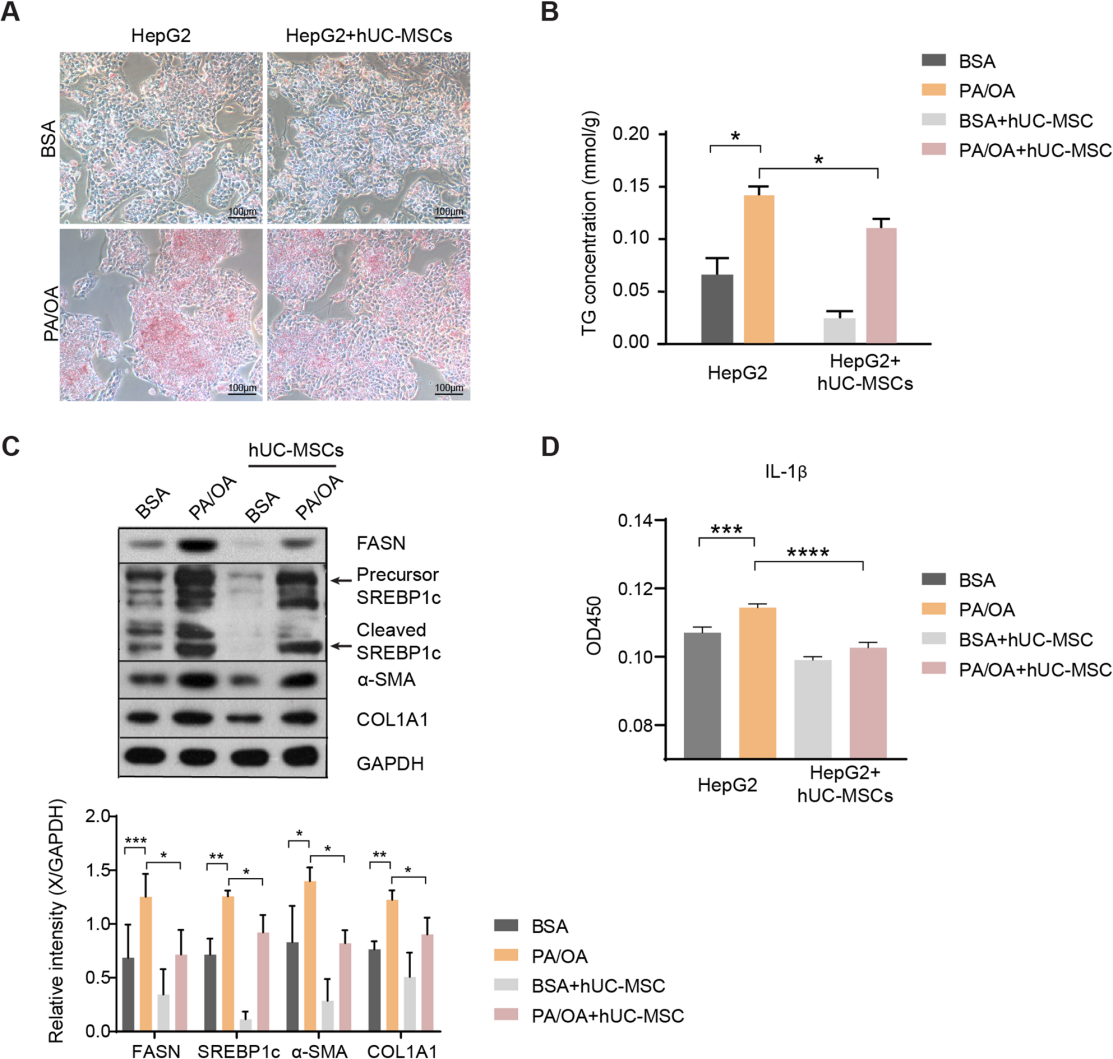
hUC-MSC treatment improved the lipid metabolism, inflammation, and fibrosis in palmitic acid and oleic acid (PA/OA)-induced HepG2 cells. HepG2 cells were stimulated by PA (100 µM) and OA (200 µM) to establish an in vitro model of NAFLD. **A** Representative Oil Red O staining of HepG2 cells pretreated with bovine serum albumin (BSA) or PA/OA for 24 h followed by treatment with or without hUC-MSCs for 12 h. Scale bar, 100 μm. **B** TG concentrations in HepG2 hepatocytes in the indicated groups. **C** The levels of lipid metabolism-associated protein (FASN and SREBP1c) and fibrosis-associated protein (α-SMA and COL1A1) in HepG2 hepatocytes. All full-length western blots are presented in Supplementary material 1: Fig. 3. **D** The secretion of the inflammatory marker IL-1β in the culture medium was detected using ELISA. Data were expressed as mean ± SD, *P < 0.05, **P < 0.01, ***P < 0.001, ****P < 0.0001

Prior literature has reported that PA/OA can induce lipid accumulation and inflammation in hepatocytes [19, 28]. To investigate the effects of hUC-MSCs on steatotic cells, an in vitro co-culture system was established using hUC-MSCs and PA/OA-induced HepG2 cells. As expected, Oil Red O staining showed that PA/OA induced aggregated lipid deposits in HepG2 cells, whereas co-culture with hUC-MSCs decreased lipid accumulation (Fig. 3A). Consistently, treatment of hUC-MSCs considerably reduced cellular TG concentration (Fig. 3B). Meanwhile, expression levels of proteins involved in fatty acid synthesis, SREBP1c and FASN, were significantly increased in PA/OA-treated HepG2 cells, but were decreased by hUC-MSCs (Fig. 3C and Supplementary Fig. 1). Similarly, fibrosis markers such as α-SMA and COL1A1 were downregulated by hUC-MSC intervention (Fig. 3C and Supplementary Fig. 1). The cytokine IL-1β promotes liver inflammation and plays a critical role in the disease progression of NAFLD [29]. We found that treatment with hUC-MSCs significantly alleviated the levels of the proinflammatory cytokine IL-1β in PA/OA-induced HepG2 cells (Fig. 3D). Overall, these results confirmed that hUC-MSCs ameliorated steatosis, inflammation, and fibrosis in PA/OA induced HepG2 cells, consistent with the in vivo findings.

### Transcriptomic analysis revealed the pathways underlying the amelioration of NAFLD following hUC-MSC infusion

**Fig. 4:**
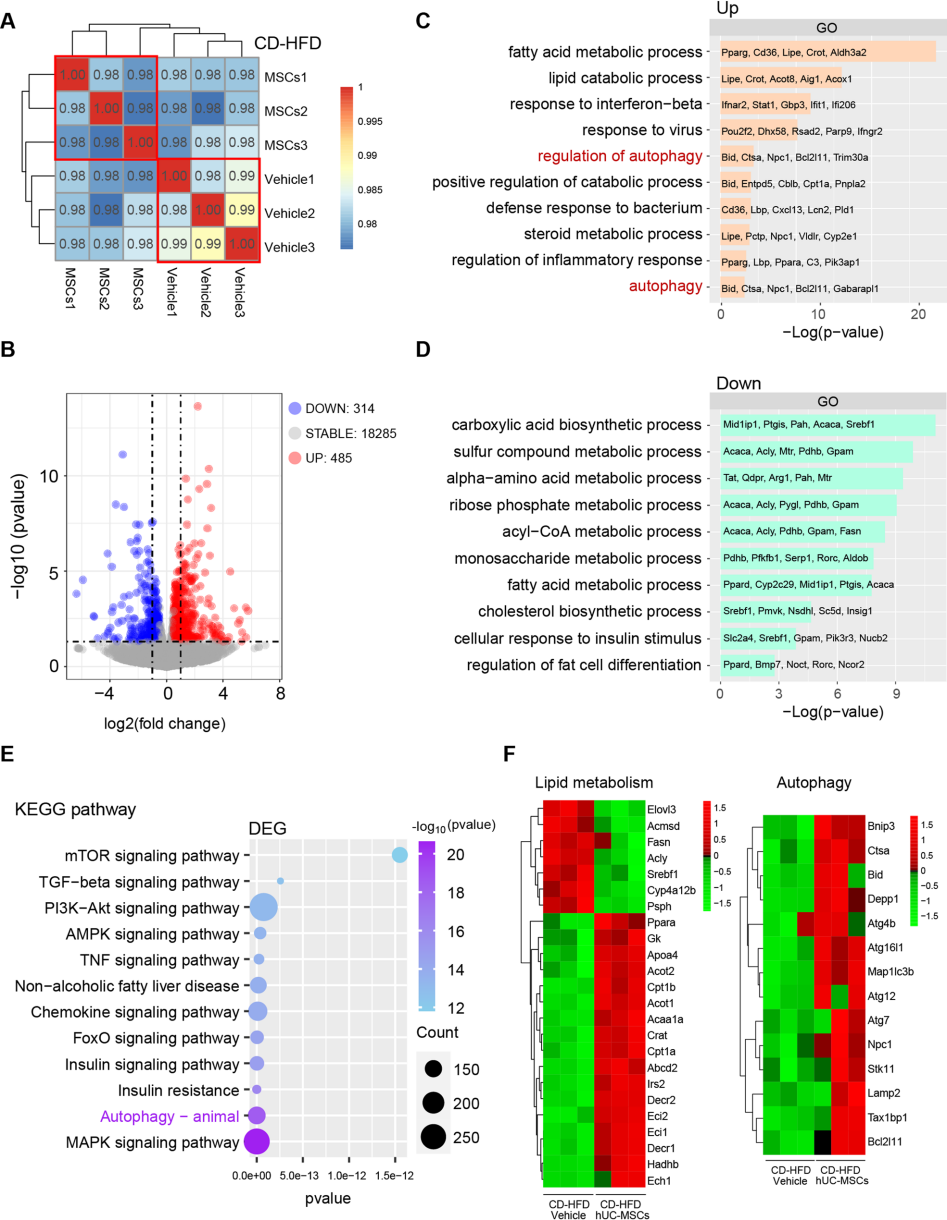
Transcriptomic profiling of liver tissues revealed molecular alterations in CD-HFD mice following hUC-MSC treatment.** A** Pairwise Pearson correlation analysis of all samples. **B** Volcano plot showing DEGs between CD-HFD hUC-MSC-treated and CD-HFD vehicle groups. Red and blue dots denote genes upregulated and downregulated in the hUC-MSC-treated group, respectively. GO enrichment analysis of genes upregulated (**C**) and downregulated (**D**) in CD-HFD mice after hUC-MSC treatment. **E** KEGG pathway enrichment analysis of DEGs between CD-HFD vehicle and CD-HFD hUC-MSC-treated groups. **F** Heatmap showing the expression of genes involved in lipid metabolism and autophagy pathways. DEGs, differentially expressed genes; GO, Gene Ontology; KEGG, Kyoto Encyclopedia of Genes and Genomes

To explore the potential molecular mechanisms by which hUC-MSCs ameliorate NAFLD, we performed transcriptomic analysis of liver tissues from CD-HFD vehicle group and CD-HFD hUC-MSCs group, using an objective, data-driven approach. Pearson correlation analysis of the transcriptomes of all mice showed that the samples in the CD-HFD vehicle group were highly consistent, and those in the hUC-MSC-treated group also exhibited strong within-group correlation. This result indicated that hUC-MSC treatment induced significant transcriptomic alterations (Fig. 4A). Subsequently, differential expression analysis identified 485 upregulated and 314 downregulated genes in the livers of hUC-MSC-treated mice compared with vehicle controls (Fig. 4B). Gene Ontology (GO) enrichment demonstrated that hUC-MSC infusion reshaped hepatic metabolic and immune pathways, indicating that hUC-MSCs may ameliorate NAFLD by suppressing lipid biosynthesis, enhancing fatty acid catabolism, promoting autophagy, and regulating inflammatory responses (Fig. 4C and D). Consistently, Kyoto Encyclopedia of Genes and Genomes (KEGG) pathway analysis showed significant enrichment of pathways associated with autophagy, energy metabolism, and inflammatory regulation (Fig. 4E). To further visualize these changes, we generated a heatmap illustrating the expression profiles of representative genes associated with lipid metabolic processes and autophagy pathways (Fig. 4F). The results revealed that hUC-MSC treatment downregulated genes related to fatty acid synthesis and lipogenesis, while upregulating genes promoting fatty acid oxidation, energy metabolism, and autophagy.

### hUC-MSCs activated autophagy in both in vivo and in vitro NAFLD models

Transcriptomic profiling revealed a significant enrichment of autophagy-related pathways (Fig. 4C and E). Given the pivotal role of autophagy in maintaining hepatic homeostasis [30] and its impairment in NAFLD pathogenesis [31–33], we next investigated whether hUC-MSC-mediated hepatoprotection was associated with autophagy activation in both in vivo and in vitro models.

**Fig. 5:**
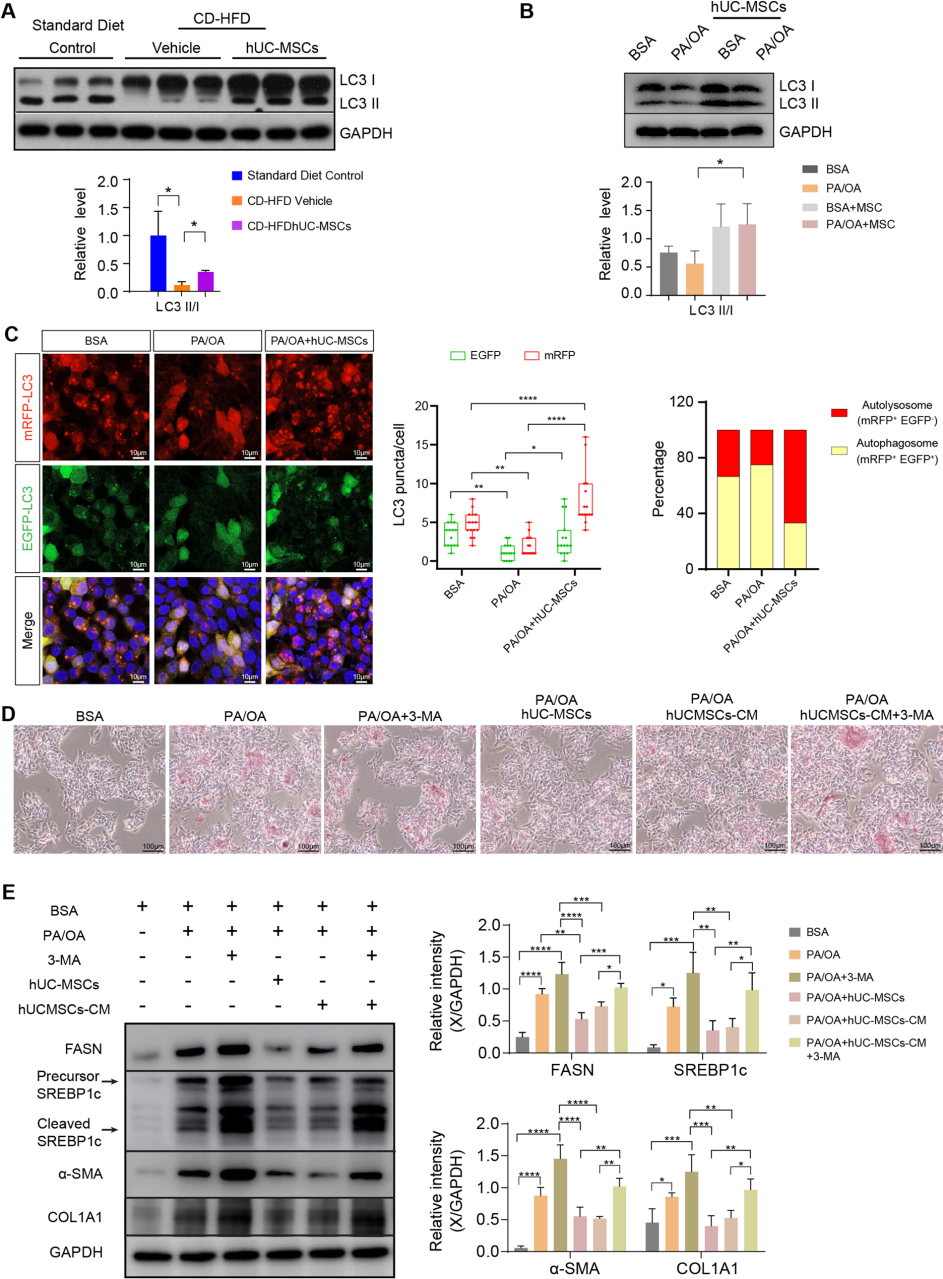
hUC-MSCs enhanced autophagic flux in NAFLD models. Western blot analysis of the levels of autophagy-associated protein (LC3 I and LC3 II) in the livers of mice (**A**) and HepG2 hepatocytes (**B**). All full-length western blots are presented in Supplementary material 1: Fig. 5. **C** Immunofluorescence analysis of autophagic flux in HepG2 cells. HepG2 cells were infected with pCDH-mRFP-EGFP-LC3 lentivirus before PA/OA treatment. Representative images showing EGFP-LC3 puncta and mRFP-LC3 puncta in HepG2 cells. Nuclei were stained with DAPI. Scale bar, 10 μm. Data was quantified by the analysis of EGFP-LC3 and mRFP-LC3 puncta. **D** Representative Oil Red O staining images of HepG2 cells in the indicated groups. After release from PA/OA treatment, HepG2 cells were treated with or without hUC-MSCs or 5 mM 3-Methyladenine (3-MA) for 12 h. hUCMSCs-CM, hUCMSCs-conditioned medium. **E** The protein levels of FASN, SREBP1c, α-SMA, and COL1A1 were detected by WB in the indicated groups. Data were expressed as mean ± SD, *P < 0.05, **P < 0.01, ***P < 0.001, ****P < 0.0001

The conversion of the soluble form of LC3 (LC3-I) to its lipidated and membrane-bound form (LC3-II), is a well-known marker of autophagosome biogenesis. We first measured the protein level of LC3 and found that the ratio of LC3II/LC3I was markedly decreased in NAFLD mice, while hUC-MSC administration upregulated LC3II/LC3I ratio, indicating that autophagy was activated by hUC-MSC treatment (Fig. 5A). Consistent with the in vivo data, hUC-MSCs also enhanced autophagy in PA/OA-treated HepG2 cells (Fig. 5B).

Next, HepG2 cells were infected with a pCDH-mRFP-EGFP-LC3B lentivirus before PA/OA treatment to examine autolysosome flux. The mRFP-EGFP-LC3B fusion protein is able to emit both green fluorescent protein (EGFP) and mRFP fluorescence. When autophagosomes fuse with acidified lysosomes, the EGFP signal is quenched while the mRFP signal remains stable. Therefore, colocalization between EGFP and mRFP denotes autophagosomes (yellow puncta), while an mRFP signal without EGFP signal denotes autolysosomes (red puncta). Fluorescence imaging (Fig. 5C) revealed that the autolysosomes/autophagosomes ratio was dramatically reduced in HepG2 cells pretreated with PA/OA, indicating impaired maturation of autophagosomes into autolysosomes. In contrast, hUC-MSCs markedly enhanced autophagic flux, as evidenced by a higher autolysosome/autophagosome ratio. To further confirm that the protection of hUC-MSCs is linked to enhanced autophagy, hUC-MSC-treated HepG2 cells were then supplemented with 3-methyladenine (3-MA), an autophagy inhibitor. Oil Red O staining of HepG2 cells showed that the therapeutic effect of hUC-MSCs on lipid deposition was attenuated by addition of 3-MA (Fig. 5D). Consistently, the hUC-MSC-induced reduction in fatty acid synthesis-related markers (SREBP1 and FASN) was reversed by 3-MA (Fig. 5E and Supplementary Fig. 2). Additionally, 3-MA partially blocked the ameliorative effect of hUC-MSCs on fibrosis (Fig. 5E and Supplementary Fig. 2). Collectively, these data demonstrated that hUC-MSCs exerted the protective effects on NAFLD via the induction of autophagy.

### hUC-MSCs induced autophagy via AMPK-mTOR-TFEB signalling pathway in both in vivo and in vitro NAFLD models

**Fig. 6:**
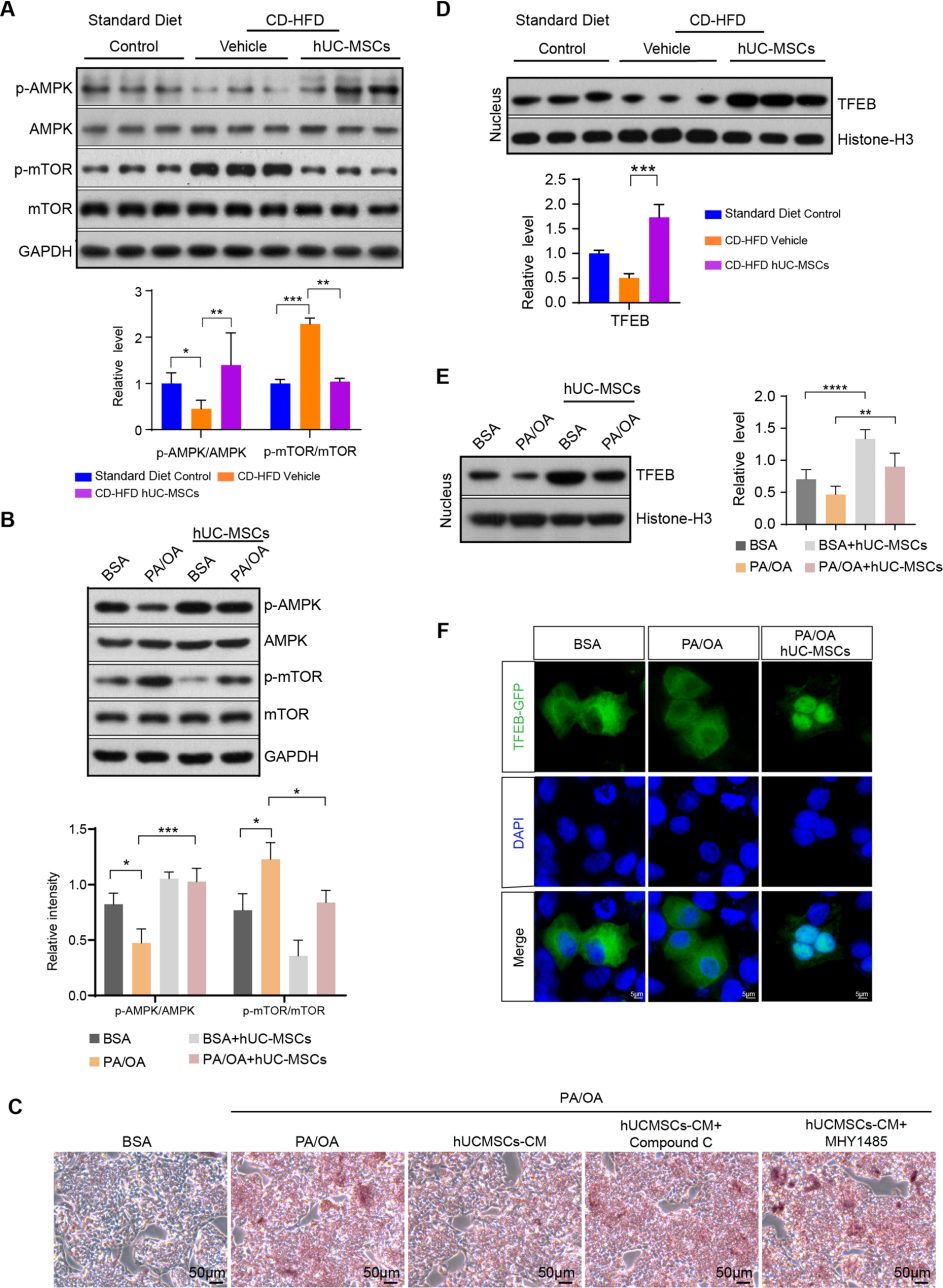
hUC-MSCs induced autophagy by regulating the AMPK-mTOR-TFEB signaling axis in the liver of mice and HepG2 cells. The protein levels of p-AMPK, AMPK, p-mTOR, and mTOR in the livers of mice (**A**) (*n* = 3 per group) and HepG2 hepatocytes (**B**) were detected by western blot. Expression levels were normalised by GAPDH. **C** Representative Oil Red O staining images of HepG2 cells in the indicated groups. After release from PA/OA treatment, HepG2 cells were treated with or without hUCMSCs-CM, 10 µM Compound C or 5 µM MHY1485 for 12 h. Compound C, an AMPK inhibitor; MHY1485, a mTOR activator. The protein levels of TFEB in the nuclei of mouse liver cells (**D**) and HepG2 cells (**E**) were measured after nucleo-cytoplasmic fractionation. Expression levels were normalised by Histone-H3. All full-length western blots are presented in Supplementary material 1: Fig. 6. **F**. Fluorescence microscopy images of HepG2 hepatocytes transfected with TFEB-GFP plasmids for each condition. Nuclei were stained with DAPI. Scale bar, 5 μm. Data were expressed as mean ± SD, *P < 0.05, **P < 0.01, ***P < 0.001, ****P < 0.0001

Transcriptomic profiling revealed that AMPK and mTOR signaling emerged as central nodes linking metabolic regulation with autophagic activity (Fig. 4E). As a key cellular energy sensor, AMPK has been reported to suppress mTOR activity [34], thereby promoting autophagy [35, 36]. Hence, the role of the AMPK-mTOR pathway in autophagy induction by hUC-MSCs was assessed. Western blots revealed that compared with NAFLD mice, phosphorylation of AMPK was remarkably enhanced in hUC-MSC treated mice, followed by suppressed phosphorylation of mTOR (Fig. 6A). In vitro data further confirmed that pre-incubation with PA/OA in HepG2 cells resulted in decreased phosphorylation of AMPK as well as activation of mTOR, whereas hUC-MSC intervention activated AMPK phosphorylation and inhibited mTOR activity (Fig. 6B). Additionally, Oil Red O staining demonstrated that treatment with the AMPK inhibitor Compound C or the mTOR activator MHY1485 partially attenuated the protective effects of hUC-MSCs against PA/OA-induced lipid accumulation in HepG2 cells (Fig. 6C). These results suggested that hUC-MSCs alleviated NAFLD and enhanced autophagy through modulation of the AMPK-mTOR signaling axis.

TFEB is a master regulator of lysosomal biogenesis and autophagy. Previous studies showed that TFEB nuclear translocation increased the transcription of genes encoding autophagic proteins and thereby promoted autophagy [37]. In addition, the subcellular localization and activity of TFEB are regulated by mTOR [38, 39]. Therefore, TFEB may serve as the downstream effector of the AMPK-mTOR pathway. To verify this hypothesis, the protein expression of TFEB in nuclear fractions was examined. Western blots showed that nuclear TFEB in NAFLD mice livers was reduced compared to standard diet control mice. However, after hUC-MSC infusion, the expression of TFEB in nuclei was remarkably increased (Fig. 6D). Consistent with in vivo findings, hUC-MSC treatment increased the decline of nuclear TFEB (Fig. 6E). Subsequently, to visualize the nuclear translocation of TFEB, immunofluorescence microscopy was employed to observe HepG2 hepatocytes transfected with TFEB-GFP plasmids. As shown in Fig. 6F, TFEB was mainly located in the cytoplasm of HepG2 cells, and administration of hUC-MSCs promoted nuclear translocation of TFEB. Altogether, the above results further reinforced the notion that hUC-MSCs induced autophagy via AMPK-mTOR-TFEB signaling pathway.

### Knockdown of TFEB impaired autophagy and attenuated the protective effect of hUC-MSCs on NAFLD

**Fig. 7:**
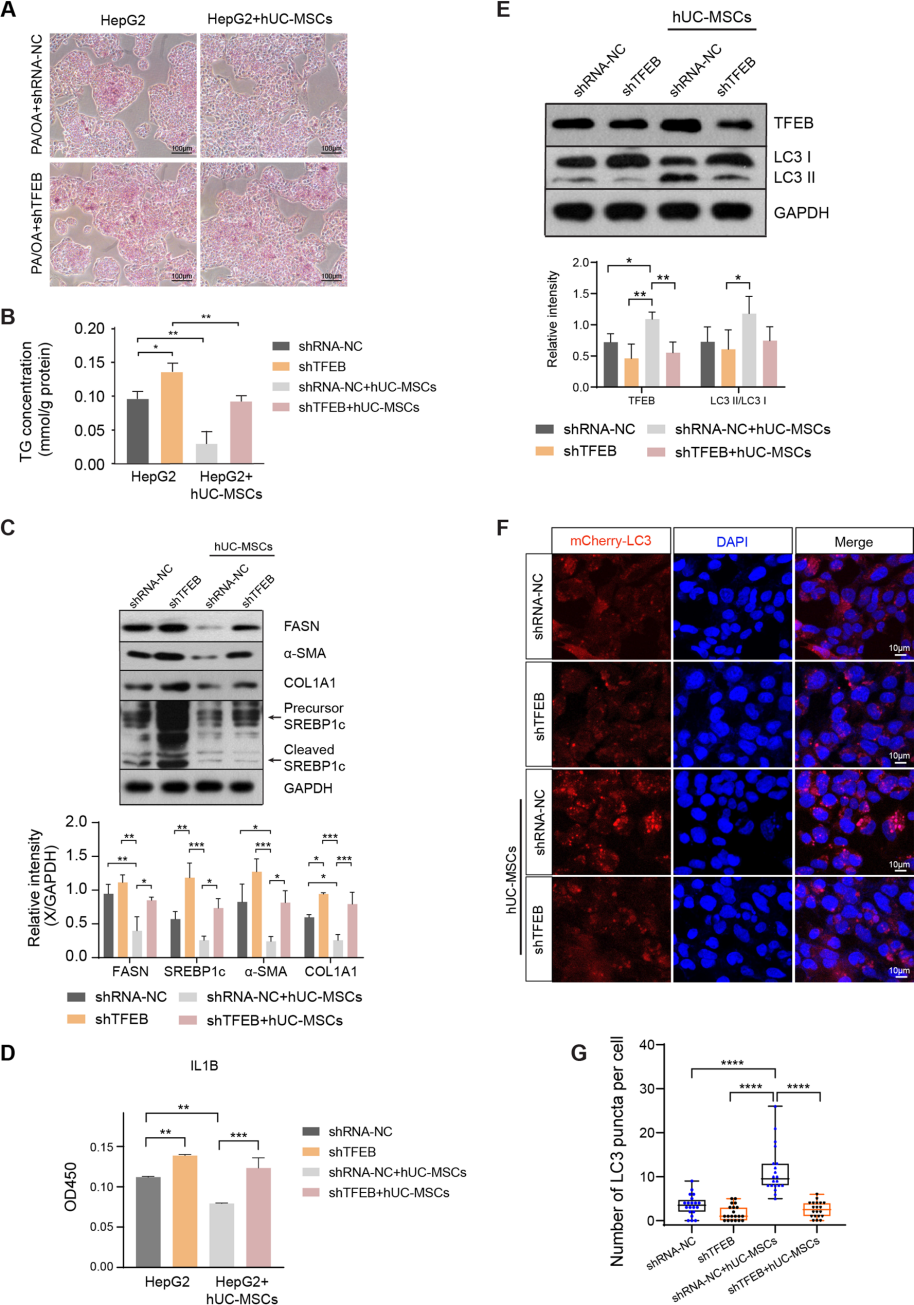
TFEB knockdown blocked hUC-MSCs-induced autophagy and impaired ameliorative effects on lipid metabolism, inflammation and fibrosis in NAFLD models.** A** Representative Oil Red O staining images of HepG2 cells in the indicated groups. **B** The TG concentrations in HepG2 cells in each group were measured. **C** The levels of lipid metabolism-associated protein (FASN and SREBP1c) and fibrosis-associated protein (α-SMA and COL1A1) in HepG2 hepatocytes. **D** The secretion of the inflammatory marker IL-1β in the culture medium was detected using ELISA. **E** The levels of autophagy-associated proteins in HepG2 cells exposed to PA/OA. All full-length western blots are presented in Supplementary material 1: Fig. 7. **F** Representative images showing mCherry-LC3 puncta in HepG2 cells after TFEB knockdown. Nuclei were stained with DAPI. Scale bar: 10 μm. **G** mCherry-LC3 (red) puncta per cell were counted. Data were expressed as mean ± SD, *p < 0.05, **p < 0.01, ***p < 0.001, and ****p < 0.0001

To further investigate whether TFEB mediated the therapeutic effect of hUC-MSCs on NAFLD, short-hairpin RNA (shRNA)-mediated knockdown of TFEB (TFEB-KD) was performed in HepG2 cells. TFEB knockdown substantially blocked the curative effect of hUC-MSCs on steatosis, as indicated by increased lipid droplets (Fig. 7A), elevated TG content (Fig. 7B), and upregulated expression of lipid metabolism-associated proteins FASN and SREBP1c (Fig. 7C). Additionally, the ameliorative effect on fibrosis was partially blocked, as fibrosis-associated proteins (α-SMA and COL1A1) were both increased by TFEB inhibition (Fig. 7C). The attenuation of proinflammatory cytokine IL-1β by hUC-MSCs was reversed after TFEB inhibition (Fig. 7D). Moreover, autophagy activation by hUC-MSC treatment was diminished in TFEB-KD HepG2 cells. The LC3II/LC3I ratio, an autophagy marker, was decreased upon TFEB knockdown (Fig. 7E). Consistently, immunofluorescence imaging demonstrated that hUC-MSC induced elevation of mCherry-LC3 puncta was impaired in TFEB-KD HepG2 cells (Fig. 7F and G). Overall, these results confirmed that hUC-MSCs ameliorated NAFLD and activated autophagy via TFEB induction.

## Discussion

**Fig. 8:**
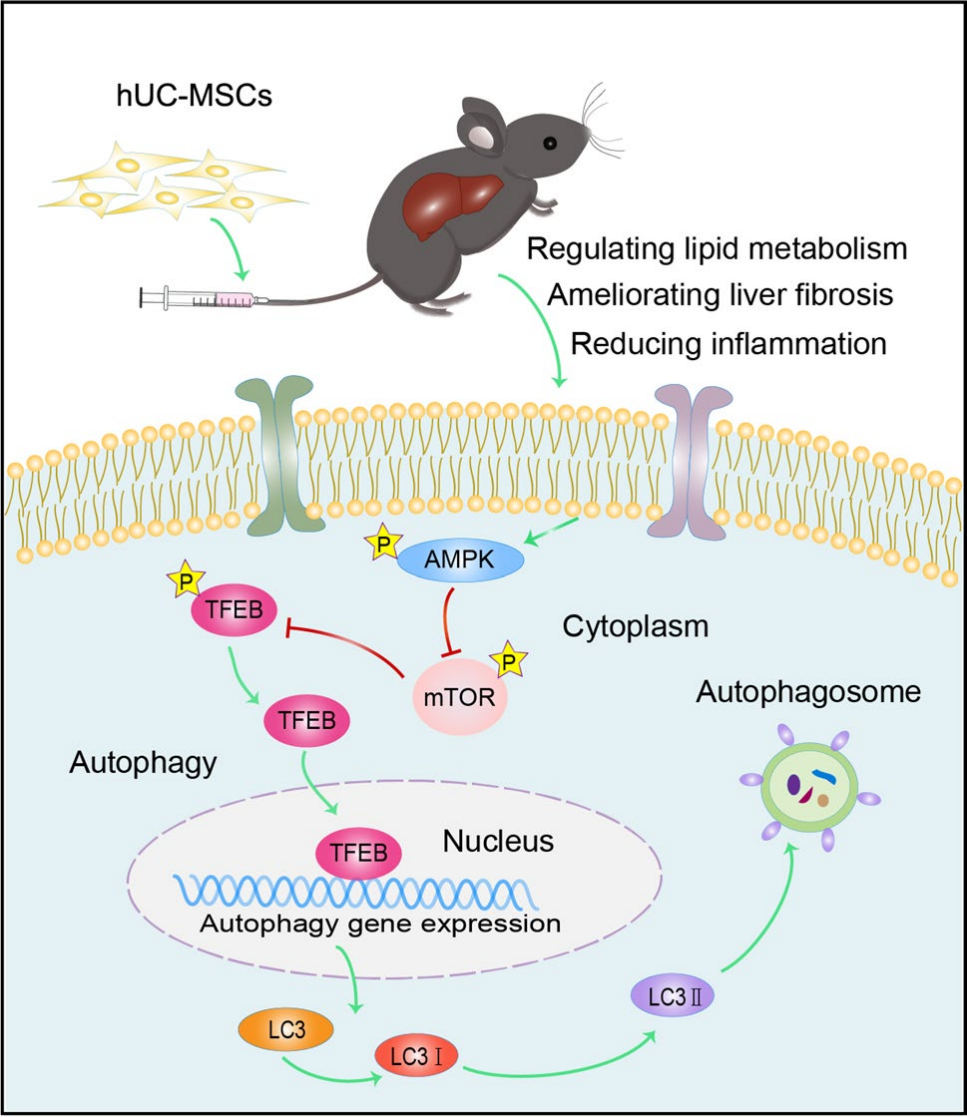
Schematic diagram of hUC-MSCs alleviating NAFLD. hUC-MSCs alleviated steatosis, inflammation and fibrosis in NAFLD via inducing autophagy, which was mediated through AMPK-mTOR-TFEB signaling pathway

In this study, hUC-MSCs exerted hepatoprotective effects by alleviating steatosis, inflammation and fibrosis in NAFLD, which was attributed to enhanced autophagy via the AMPK-mTOR-TFEB signaling axis (Fig. 8).

The pathogenesis and progression of NAFLD are considered to be a complex and multifactorial process that remain incompletely understood. Autophagy, a lysosome-dependent cellular degradation process, has been implicated as a vital regulatory factor in the development of NAFLD [40]. Published data have suggested that mice with autophagy-related 5 (Atg5) deficiency in liver CD11c + cells exhibit increased TG accumulation in liver tissues and elevated serum levels of alanine aminotransferase [41]. Here, both autophagy marker LC3B-II and autophagic flux were reduced in NAFLD mice and in PA/OA-induced HepG2 hepatocytes. This finding is consistent with previous clinical data, which revealed impaired autophagy in liver biopsy specimens from NAFLD patients [41]. Additionally, it is worth noting that autophagy induction by pharmacological treatments, such as ezetimibe and curcumin, have been shown to protect against hepatic steatosis and injury [42, 43]. Thus, given its essential role in maintaining hepatic homeostasis, autophagy activation represents a potential therapeutic target for NAFLD [30].

Current therapeutic strategies for NAFLD, including lifestyle and pharmacological interventions, encounter significant limitations. Lifestyle modifications, such as diet and exercise, are fundamental but often hindered by poor long-term adherence [44, 45]. Pharmacological agents, including pioglitazone and SGLT2 inhibitors, have shown potential in reducing hepatic steatosis and improving metabolic parameters, yet their long-term safety and efficacy in reversing fibrosis remain uncertain [46, 47]. Resmetirom, a liver-selective thyroid hormone receptor-beta agonist, has received conditional approval for treating NASH patients with fibrosis stages F2-F3 [5]. However, clinical trials have indicated that only 30% of patients achieved NASH resolution without fibrosis progression, with even fewer attaining a one-stage fibrosis improvement. GLP-1 receptor agonists (GLP-1RAs) effectively promote weight loss, reduce liver injury, and induce steatohepatitis resolution, though their impact on fibrosis, particularly in advanced disease, remains inconclusive [48, 49]. Dual and triple incretin agonists exhibit superior metabolic benefits, yet their effects on NASH and fibrosis are still unverified [50]. Additionally, gastrointestinal adverse effects associated with these therapies may limit patient tolerability. These challenges highlight the critical need for innovative therapeutic approaches that target the multifactorial pathogenesis of NAFLD.

The hUC-MSCs are self-renewing multipotent cells that have attracted increasing attention for treating a variety of diseases owing to their non-invasive accessibility [51, 52]. Recently, transplantation of hUC-MSCs has also emerged as a promising therapeutic option for NAFLD [8–10]. Previous studies have indicated that hUC-MSCs can protect against liver steatosis, inflammation and fibrosis [9, 10]. Furthermore, hUC-MSC derived exosomes have been proven to ameliorate insulin resistance, lipid accumulation as well as oxidative stress [53–55]. Consistent with these findings, our study confirmed the therapeutic potential of hUC-MSCs, as evidenced by reductions in hepatic lipid droplets and the attenuation of inflammatory cytokines and transaminases. Compared to existing therapeutic strategies, the advantage of hUC-MSCs in treating NAFLD lies in their ability to concurrently address lipid dysregulation, inflammatory responses, and fibrosis, which are the core pathological drivers of the disease’s complex pathogenesis. However, in our study, MSC treatment did not lead to a reduction in body weight among NAFLD mice. Notably, previous studies have reported inconsistent effects of MSC treatment on body weight, highlighting the complexity of evaluating MSC-mediated therapeutic outcomes. For instance, a single injection of 1.5 × 10^6^ hUC-MSCs reduced body weight in WD-induced NAFLD mice [10]. Similarly, two doses of bone marrow mesenchymal stem cells (BM-MSCs) at 1 × 10^7^ cells/kg significantly mitigated HFD-induced weight gain in T2DM-associated NAFLD mice [56]. In contrast, weekly injections of 1.0 × 10^6^ hUC-MSCs for six weeks had no effect on the body weight of HFD-fed mice [57]. Likewise, two doses of 0.5 × 10^6^ syngeneic BM-MSCs failed to reverse metabolic syndrome or obesity in obese mice with metabolic syndrome [58]. These discrepancies may arise from factors such as variations in feed formulations, MSC biological properties (which are influenced by donor characteristics, isolation methods, and culture conditions) [59, 60], differences in MSC dosages and treatment frequencies, and variations in the timing of weight measurements. Given that our study focused on a defined short- to mid-term observation window, future investigations with extended observation periods and longitudinal assessments will be valuable to further characterize the durability and long-term efficacy of hUC-MSCs in NAFLD.

However, the mechanism by which hUC-MSCs exert protective effects against NAFLD remains to be elucidated. Previous studies have demonstrated that MSCs play a critical role in modulating the microenvironment and regulating both local and systemic inflammation. The paracrine potential of MSCs, including the secretion of microRNAs and cytokines, is pivotal to these processes [11, 53, 61]. Intravenous transplantation of UC-MSC exosomes into MCD-induced NASH mice led to the recruitment of more macrophages to the liver, suggesting that MSC exosomes may alter the distribution and chemotaxis of macrophages in the damaged liver. Furthermore, they effectively polarized macrophages toward the anti-inflammatory M2 phenotype and ameliorated MCD-induced liver injury in mice [61]. A recent study further demonstrated that MSCs can promote the production of IL-10 by regulatory T cells in the spleen, both in vivo and in vitro, thereby promoting macrophage polarization and improving systemic metabolic homeostasis in HFD mice [57]. Additionally, MSCs exhibit immunomodulatory properties by reducing the proportion of CD4^+^ IFN-γ^+^ and CD4^+^ IL-6^+^ T cells in the spleen, which contributes to improved liver function and reduced inflammation [62]. In this study, we confirmed that hUC-MSCs reduced the levels of proinflammatory cytokines (IL-6, IL-1β, and TGF-β1) in CD-HFD-induced NAFLD mice, both in the liver and in the serum. This effect may be related to the distribution of intravenously infused hUC-MSCs in the spleens and lungs, in addition to the liver. However, the underlying and intricate immune regulatory mechanisms remain to be fully explored with a large number of additional experiments involving local perturbations of MSCs, which could be conducted in the futures. Overall, existing research has clarified the effects of MSCs mainly from the perspective of immune modulation and paracrine secretion, while few studies have explored the interactions between hUC-MSCs and autophagy in NAFLD.

The modulation of autophagy by MSCs has been implicated in other liver or kidney diseases, including CCl4-induced liver fibrosis [63], cholestatic liver disease [64], hepatic ischemia-reperfusion injury [65], and diabetic nephropathy [66]. In cases of cholestatic liver disease, BM-MSCs mitigated the activation of hepatic stellate cells (HSCs) through the regulation of autophagy via the PI3K/AKT/mTOR pathway [64]. Additionally, a study investigating liver ischemia/reperfusion injury revealed that BM-MSCs exerted a therapeutic effect through heme oxygenase‑1-induced autophagy. Furthermore, recent investigations have highlighted the potential of exosomes derived from human BM-MSCs, which contain let-7a-5p, in promoting autophagy by targeting MAP4K3 in models of acute-on-chronic liver failure [67]. Similarly, in kidney disorders such as diabetic nephropathy, exosomes from adipose-derived stem cells have been shown to enhance autophagy flux, alleviate podocyte injury and improve symptoms in type 2 diabetic mice by inhibiting mTOR signaling activation. Therefore, these findings suggest that regulation of autophagy could be a potential mechanism underlying the therapeutic effect of MSCs. In the present study, the downregulation of autophagy markers and autophagic flux in NAFLD models was restored by hUC-MSC treatment, along with the improvement of hepatic steatosis, inflammation and fibrosis. Hence, these results illustrated the association of the hepatoprotective effects of hUC-MSCs with the induction of autophagy.

TFEB is a positive transcriptional regulator of autophagy that functions by triggering the transcription of autophagy-related genes [68]. TFEB shuttles between the cytoplasm and the nucleus through regulation of its phosphorylation sites [16]. Studies have shown that the expression and cellular distribution of TFEB was associated with the pathogenesis of NAFLD. The nuclear levels of TFEB were inversely correlated with the steatosis scores of liver biopsy samples from NAFLD patients [17]. In the present study, nuclear expression of TFEB was decreased in NAFLD mice compared with the standard diet control group, which was further validated in the HepG2 cell line model of NAFLD. Notably, hUC-MSC administration promoted TFEB translocation to the nucleus and enhanced the autophagic flux in both in vitro and in vivo experiments. Furthermore, knockdown of TFEB in HepG2 cells reduced the therapeutic effects of hUC-MSCs as well as the activation of autophagy. Taken together, to the best of our knowledge, this study is the first to demonstrate that hUC-MSCs improved NAFLD symptoms through TFEB-dependent autophagy.

It is noteworthy that the molecular mechanism by which hUC-MSCs accelerated TFEB nuclear translocation and induced autophagy remains unclear. To address this, the main regulator and signaling pathway involved in the curative effects of hUC-MSCs were further investigated. The AMPK-mTOR pathway has been reported to play a critical role in regulating autophagy and coordinating cellular homeostasis in response to stress [69, 70]. It has been recognized that mTOR acts as a master regulator of TFEB and induces its cytoplasmic retention under nutrient-replete conditions, thereby inhibiting autophagy [71]. In the present study, the administration of hUC-MSCs inhibited mTOR activation and promoted the nuclear translocation of TFEB. As an upstream repressor of mTOR, AMP-activated protein kinase (AMPK) inhibits the activation of mTOR, leading to nuclear translocation of TFEB and autophagy induction, thereby coordinating autophagy and maintaining energy homeostasis. The interactions between AMPK and mTOR have been well elucidated. Existing literature suggested that AMPK represses mTOR through the activation of tuberous sclerosis complex 2 as well as phosphorylation of mTOR binding partner raptor [35, 36]. Therefore, AMPK-dependent inhibition of mTOR is a well-recognized activator of autophagy. Depressed AMPK and hyperactivation of mTOR have been coupled with NAFLD [72], which was also confirmed in this study. Of note, previous studies have demonstrated that hUC-MSCs were involved in the regulation of AMPK-mTOR signaling. It has been reported that hUC-MSCs improved high glucose-induced rat podocyte injuries in diabetic nephropathy rats via the AMPK-mTOR pathway [73]. Consistently, the present work revealed that infusion of hUC-MSCs promoted AMPK activation and suppressed mTOR. Therefore, it is plausible that hUC-MSCs alleviated NAFLD and promoted autophagy through AMPK-mTOR-TFEB signaling pathway.

Emerging evidence indicates that MSC-derived exosomes play a crucial role in regulating autophagy across various disease models [67, 74–76]. Lin et al. demonstrated that MSC-derived exosomes enriched with let-7a-5p directly target MAP4K3 in hepatocytes, reducing TFEB phosphorylation and promoting its nuclear translocation to activate autophagy-related genes (e.g., LC3, Beclin-1) and restore autophagic flux in acute-on-chronic liver failure models [67]. Additionally, exosomes containing miR-486 secreted by adipose-derived stem cells (ADSCs) have been shown to enhance autophagic flux in podocytes by inhibiting Smad1/mTOR signaling, thereby ameliorating diabetic nephropathy [77]. These findings indicate that MSCs may indirectly regulate autophagy in target cells within damaged tissues by balancing pro-survival and stress-response pathways through exosomes. Furthermore, the paracrine effects of MSCs represent a pivotal mechanism for orchestrating autophagy in recipient cells. A recent study revealed that the ADSC secretome achieved comparable therapeutic efficacy to transplanted ADSCs in mitigating hepatic ischemia-reperfusion injury and modulating hepatocyte autophagy, suggesting that paracrine signaling is primarily responsible for these effects [65]. While our current study primarily focuses on the overall effects of MSCs on hepatocyte autophagy, future research should leverage multi-omics technologies for a more comprehensive investigation of the underlying mechanisms.

## Conclusion

In summary, this study demonstrates that administration of hUC-MSCs alleviates steatosis, inflammation and fibrosis in NAFLD via inducing autophagy, which is mediated through the AMPK-mTOR-TFEB signaling pathway. These findings suggest that hUC-MSCs may serve as a novel therapeutic approach for the treatment of NAFLD.

## Supplementary Information

Below is the link to the electronic supplementary material.

Supplementary Material 1

Supplementary Material 2

Supplementary Material 3

Supplementary Material 4

## Data Availability

The bulk RNA-seq data collected in this study are provided in Supplementary Material 4. All data generated or analyzed during this study are included in the main article and its supplementary files. Lead contact. Congrong Wang(crwang@tongji.edu.cn). The materials included in this study are available from the lead contact upon reasonable request.
