# Supplementary material for: Human umbilical cord-derived mesenchymal stem cells ameliorate non-alcoholic fatty liver disease via activating TFEB-mediated autophagy in male mice
Source: Stem Cell Res Ther. 2025 Dec 13;17:34. doi: 10.1186/s13287-025-04855-9 (PMC12817827; doi:10.1186/s13287-025-04855-9)
Supplement: Supplementary file 1 — Supplementary Material 1 [file 13287_2025_4855_MOESM1_ESM.docx]

**Original western blot gels**

**Figure 2**

| **Figure 2F** | **FASN**  **273 kDa** | 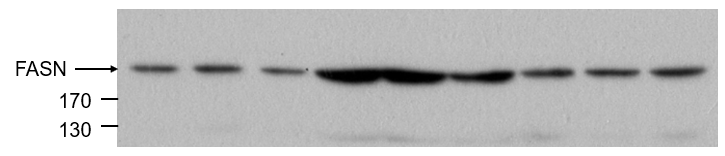 |
| --- | --- | --- |
| **Figure 2F** | **GAPDH**  **36 kDa** | 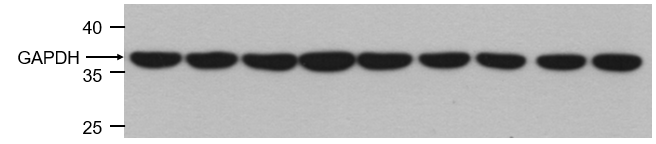 |
| **Figure 2F** | **SREBP1c**  **precursor ~120 kDa**  **cleaved ~68 kDa** | 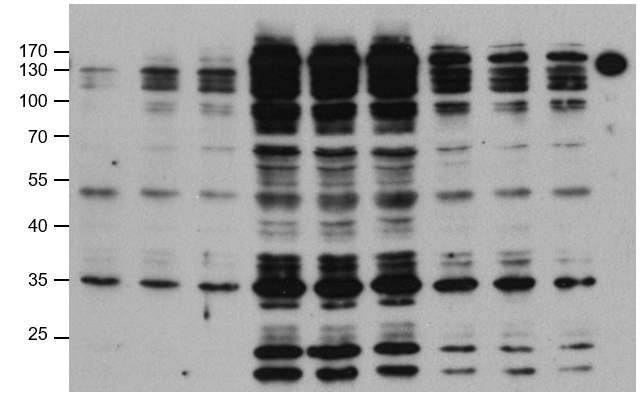 |
| **Figure 2F** | **GAPDH**  **36 kDa** | 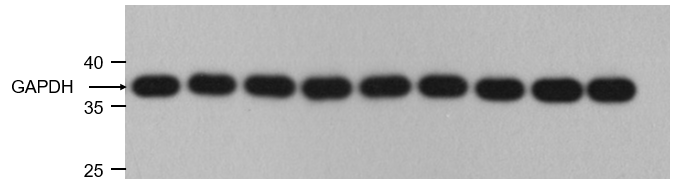 |
| **Figure 2K** | **a-SMA**  **42 kDa** | 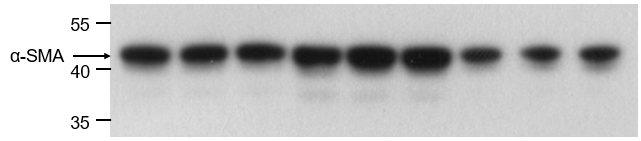 |
| **Figure 2K** | **COL1A1**  **120-130 kDa** | 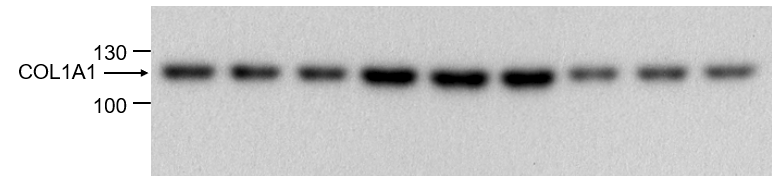 |
| **Figure 2K** | **GAPDH**  **36 kDa** | 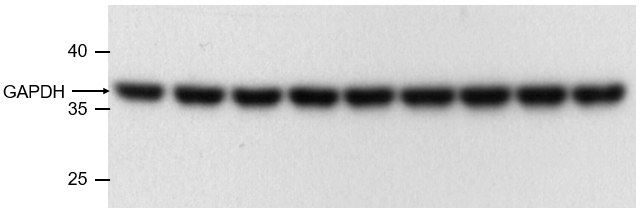 |

**Figure 3**

| **Figure 3C** | **FASN**  **273 kDa** | 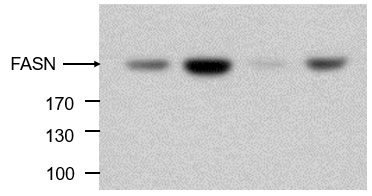 |
| --- | --- | --- |
| **Figure 3C** | **SREBP1c**  **precursor ~120 kDa**  **cleaved ~68 kDa** | 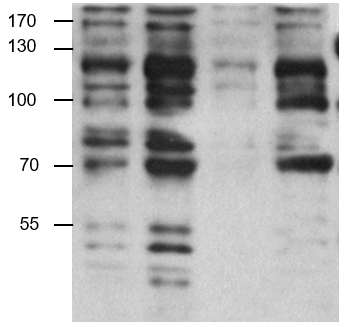 |
| **Figure 3C** | **a-SMA**  **42 kDa** | 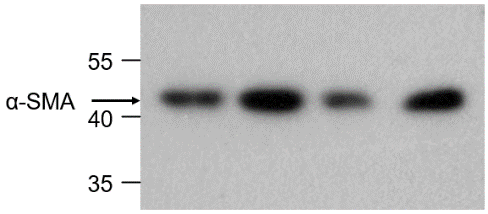 |
| **Figure 3C** | **COL1A1**  **120-130 kDa** | 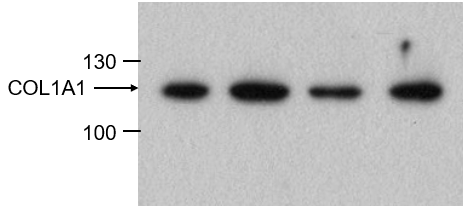 |
| **Figure 3C** | **GAPDH**  **36 kDa** | 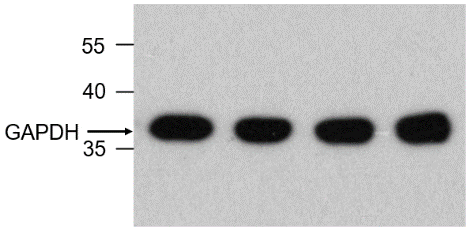 |

**Figure 5**

| **Figure 5A** | **LC3B**  **LC3 I 16 kDa**  **LC3 II 14 kDa** | 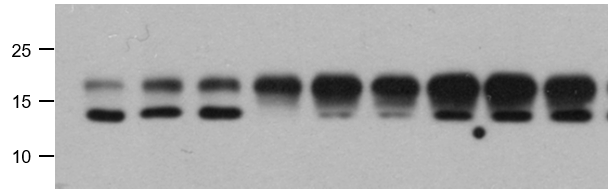 |
| --- | --- | --- |
| **Figure 5A** | **GAPDH**  **36 kDa** | 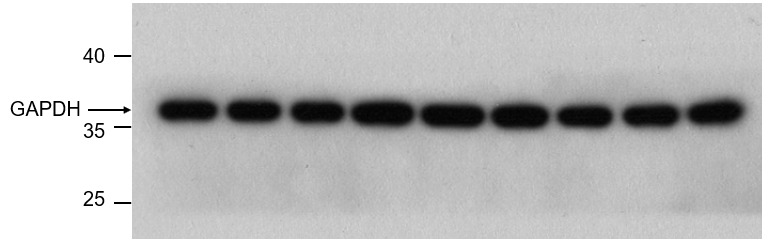 |
| **Figure 5B** | **LC3B**  **LC3 I 16 kDa**  **LC3 II 14 kDa** | 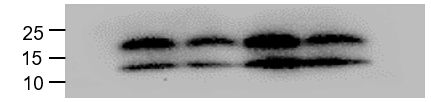 |
| **Figure 5B** | **GAPDH**  **36 kDa** | 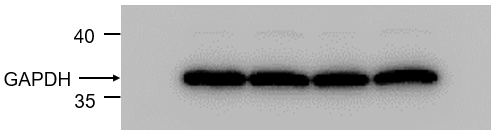 |
| **Figure 5E** | **FASN**  **273 kDa** | 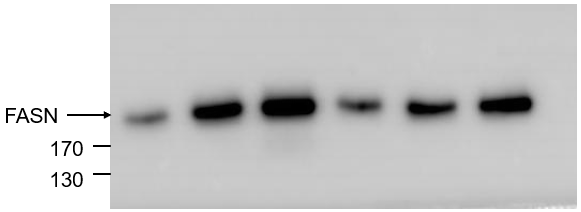 |
| **Figure 5E** | **SREBP1c**  **precursor ~120 kDa**  **cleaved ~68 kDa** | 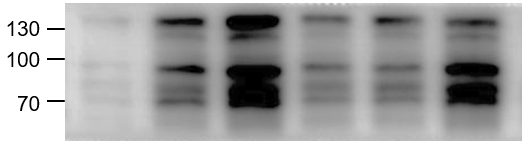 |
| **Figure 5E** | **a-SMA**  **42 kDa** | 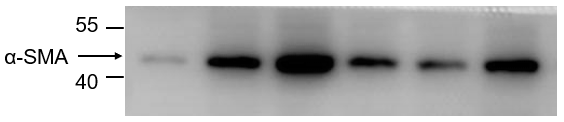 |
| **Figure 5E** | **COL1A1**  **120-130 kDa** | 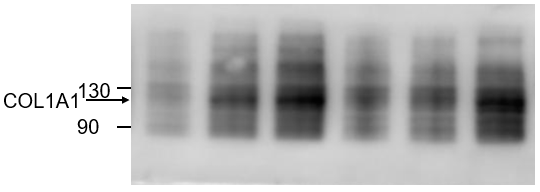 |
| **Figure 5E** | **GAPDH**  **36 kDa** | 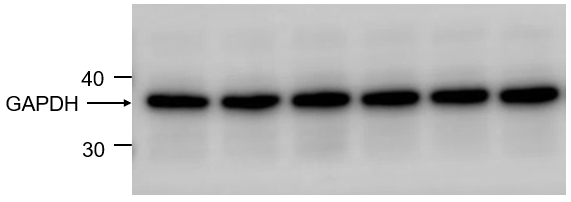 |

**Figure 6**

| **Figure 6A** | **p-AMPK**  **62 kDa** | 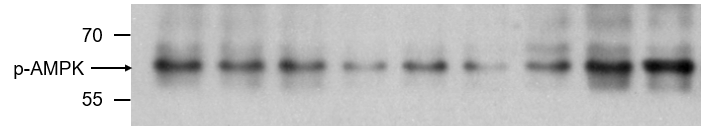 |
| --- | --- | --- |
| **Figure 6A** | **AMPK**  **62 kDa** | 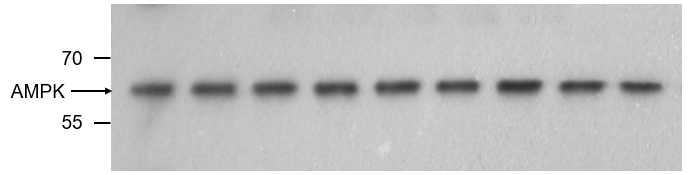 |
| **Figure 6A** | **p-mTOR**  **289 kDa** | 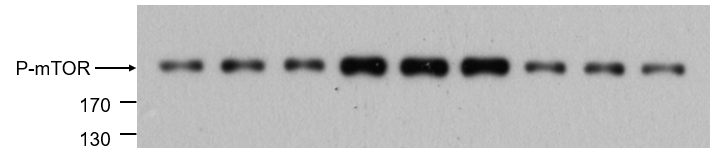 |
| **Figure 6A** | **mTOR**  **289 kDa** | 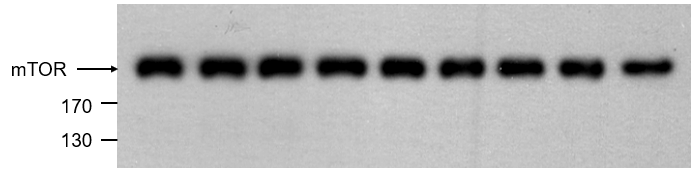 |
| **Figure 6A** | **GAPDH**  **36 kDa** | 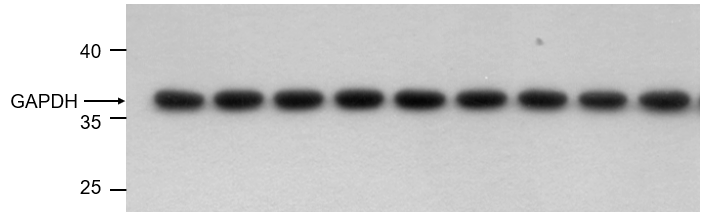 |
| **Figure 6B** | **p-AMPK**  **62 kDa** | 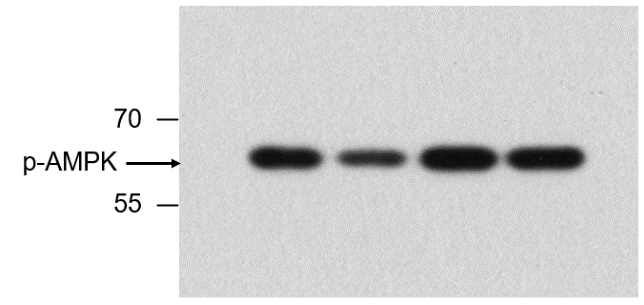 |
| **Figure 6B** | **AMPK**  **62 kDa** | 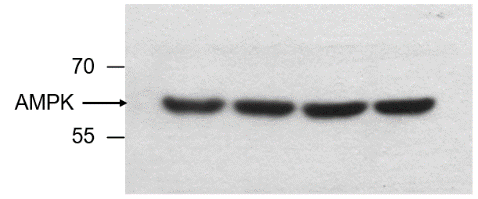 |
| **Figure 6B** | **p-mTOR**  **289 kDa** | 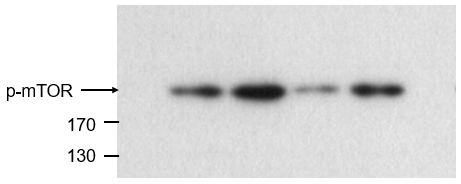 |
| **Figure 6B** | **mTOR**  **289 kDa** | 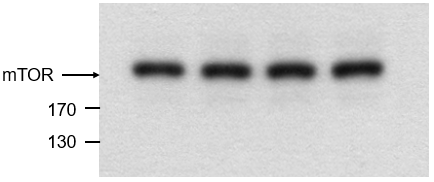 |
| **Figure 6B** | **GAPDH**  **36 kDa** | 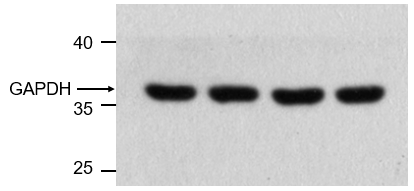 |
| **Figure 6D** | **TFEB**  **65-70 kDa** | 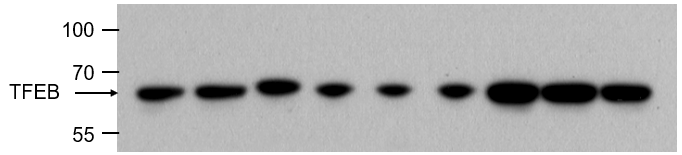 |
| **Figure 6D** | **Histone-H3**  **17 kDa** | 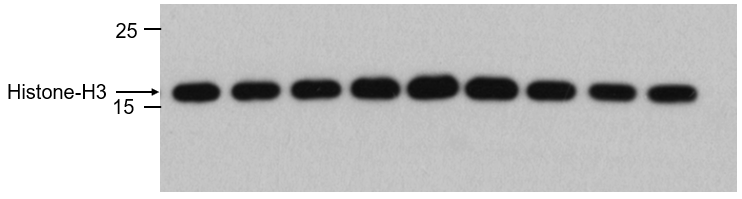 |
| **Figure 6E** | **TFEB**  **65-70 kDa** | 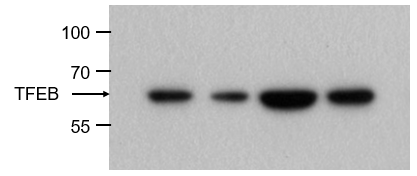 |
| **Figure 6E** | **Histone-H3**  **17 kDa** | 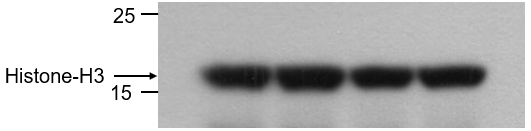 |

**Figure 7**

| **Figure 7C** | **FASN**  **273 kDa** | 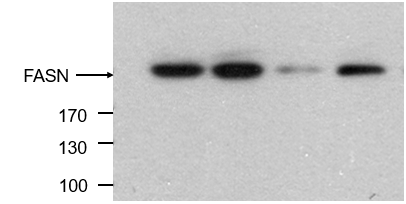 |
| --- | --- | --- |
| **Figure 7C** | **a-SMA**  **42 kDa** | 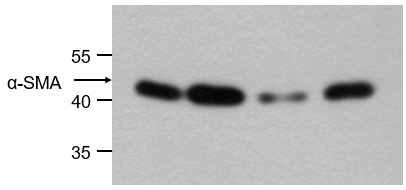 |
| **Figure 7C** | **SREBP1c**  **precursor ~120 kDa**  **cleaved ~68 kDa** | 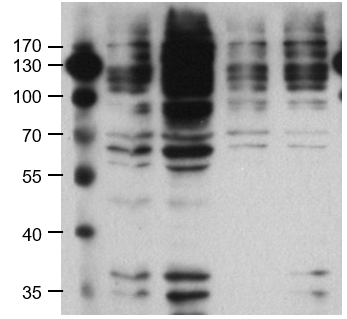 |
| **Figure 7C** | **COL1A1**  **120-130 kDa** | 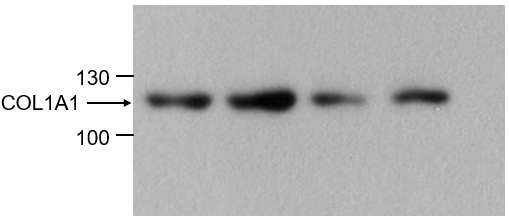 |
| **Figure 7C** | **GAPDH**  **36 kDa** | 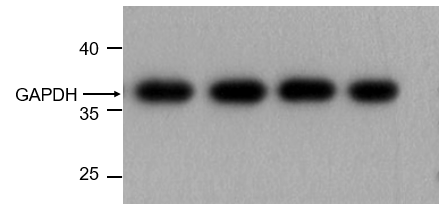 |
| **Figure 7E** | **TFEB**  **65-70 kDa** | 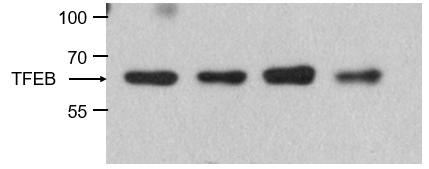 |
| **Figure 7E** | **LC3B**  **LC3 I 16 kDa**  **LC3 II 14 kDa** | 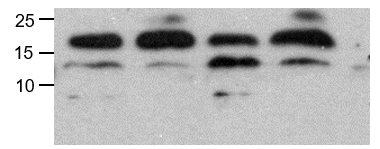 |
| **Figure 7E** | **GAPDH**  **36 kDa** | 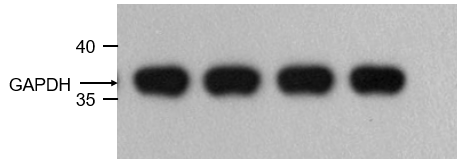 |
