# Supplementary material for: Human umbilical cord-derived mesenchymal stem cells ameliorate non-alcoholic fatty liver disease via activating TFEB-mediated autophagy in male mice
Source: Stem Cell Res Ther. 2025 Dec 13;17:34. doi: 10.1186/s13287-025-04855-9 (PMC12817827; doi:10.1186/s13287-025-04855-9)
Supplement: Supplementary file 2 — Supplementary Material 2 [file 13287_2025_4855_MOESM2_ESM.docx]

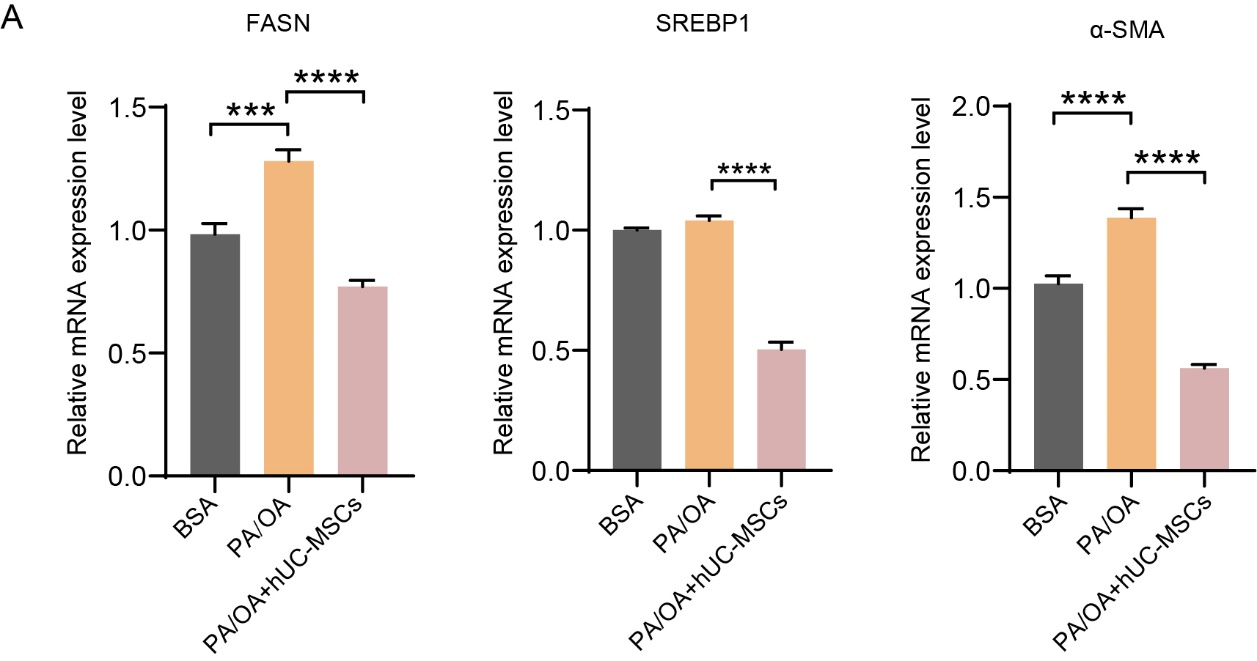
**Supplementary Figure 1**

**Figure S1** HepG2 cells were pretreated with bovine serum albumin (BSA) or PA/OA for 24 h followed by treatment with or without hUC-MSCs for 12 h. The mRNA levels of FASN, SREBP1 and α-SMA were detected by qRT-PCR in the indicated groups. Data were expressed as mean ± SD, ****P* < 0.001, *****P* < 0.0001.


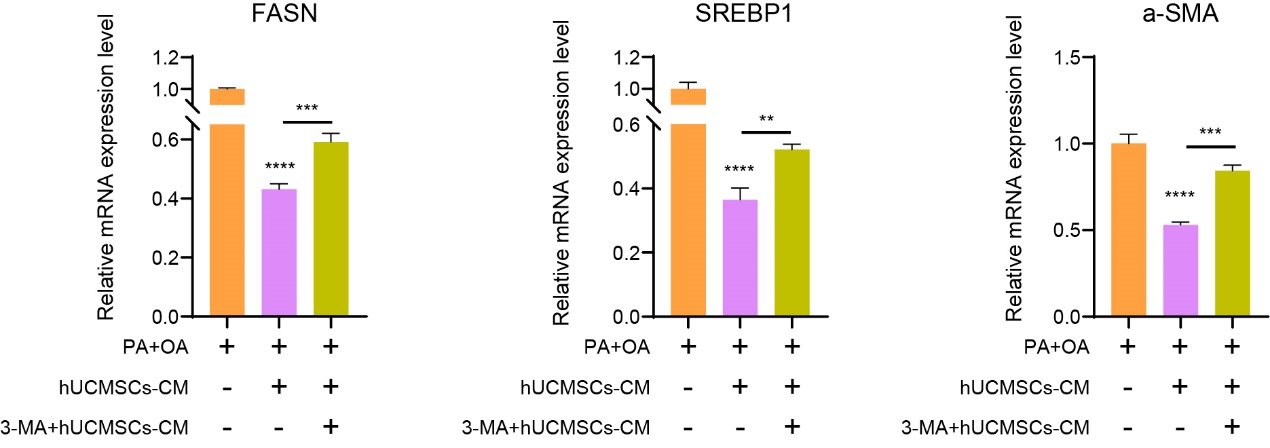
**Supplementary Figure 2**

**Figure S2** After release from PA/OA treatment, HepG2 cells were treated with or without hUCMSC-CM or 3-Methyladenine (3-MA) for 12 h. The mRNA levels of FASN, SREBP1 and α-SMA were detected by qRT-PCR in the indicated groups. hUCMSCs-CM, hUCMSCs-conditioned medium. Data were expressed as mean ± SD, **P < 0.01, ***P < 0.001, ****P < 0.0001.
