# Supplementary material for: Human umbilical cord-derived mesenchymal stem cells ameliorate non-alcoholic fatty liver disease via activating TFEB-mediated autophagy in male mice
Source: Stem Cell Res Ther. 2025 Dec 13;17:34. doi: 10.1186/s13287-025-04855-9 (PMC12817827; doi:10.1186/s13287-025-04855-9)
Supplement: Supplementary file 3 — Supplementary Material 3 [file 13287_2025_4855_MOESM3_ESM.docx]

**Supplementary Table 1**

**Table S1. Primer sequences used in this study**

| **Target gene** | **Forward primer (5'--3')** | **Reverse primer (5'--3')** |
| --- | --- | --- |
| Mouse-Srebp1 | GGAGCCATGGATTGCACATT | GGCCCGGGAAGTCACTGT |
| Mouse-α-SMA | CCCAGACATCAGGGAGTAATGG | TCTATCGGATACTTCAGCGTCA |
| Mouse-Col1a1 | GCTCCTCTTAGGGGCCACT | CCACGTCTCACCATTGGGG |
| Mouse-Il1β | GCAACTGTTCCTGAACTCAACT | ATCTTTTGGGGTCCGTCAACT |
| Mouse-Il6 | TCTATACCACTTCACAAGTCGGA | GAATTGCCATTGCACAACTCTTT |
| Mouse-Tgfβ1 | CCACCTGCAAGACCATCGAC | CTGGCGAGCCTTAGTTTGGAC |
| Mouse-Fasn | GGAGGTGGTGATAGCCGGTAT | TGGGTAATCCATAGAGCCCAG |
| Mouse-Gapdh | AGGTCGGTGTGAACGGATTTG | GGGGTCGTTGATGGCAACA |
| Human-FASN | AAGGACCTGTCTAGGTTTGATGC | TGGCTTCATAGGTGACTTCCA |
| Human-SREBP1 | GGAGGGGTAGGGCCAACGGCCT | CATGTCTTCGAAAGTGCAATCC |
| Human-α-SMA | GTGTTGCCCCTGAAGAGCAT | GCTGGGACATTGAAAGTCTCA |
| Human-GAPDH | GGAGCGAGATCCCTCCAAAAT | GGCTGTTGTCATACTTCTCATGG |
